# Molnupiravir clinical trial simulation suggests that polymerase chain reaction underestimates antiviral potency against SARS-CoV-2

**DOI:** 10.1172/JCI192052

**Published:** 2025-08-28

**Authors:** Shadisadat Esmaeili, Katherine Owens, Ugo Avila-Ponce de Leon, Joseph F. Standing, David M. Lowe, Shengyuan Zhang, James A. Watson, William H.K. Schilling, Jessica Wagoner, Stephen J. Polyak, Joshua T. Schiffer

**Affiliations:** 1 Vaccine and Infectious Disease Division, Fred Hutchinson Cancer Center, Seattle, Washington, USA.; 2 Infection, Immunity and Inﬂammation, Great Ormond Street Institute of Child Health, University, College London, London, United Kingdom.; 3 Great Ormond Street Hospital for Children NHS Trust, London, United Kingdom.; 4 Department of Clinical Immunology, Royal Free London NHS Foundation Trust, London, United Kingdom.; 5 Institute of Immunity and Transplantation, University College London, London, United Kingdom.; 6 Infectious Diseases Data Observatory, Oxford, United Kingdom.; 7 Centre for Tropical Medicine and Global Health, Nuffield Department of Medicine, University of Oxford, Oxford, United Kingdom.; 8 Mahidol Oxford Tropical Medicine Research Unit, Bangkok, Thailand.; 9 Department of Laboratory Medicine & Pathology, and; 10 Department of Medicine, University of Washington, Seattle, Washington, USA.

**Keywords:** Immunology, Infectious disease, Virology, COVID-19

## Abstract

Molnupiravir is an antiviral medicine that induces lethal copying errors during SARS-CoV-2 RNA replication. Molnupiravir reduced hospitalization in one pivotal trial by 50% and had variable effects on reducing viral RNA levels in three separate trials. We used mathematical models to simulate these trials and closely recapitulated their virologic outcomes. Model simulations suggested lower antiviral potency against pre-Omicron SARS-CoV-2 variants than against Omicron. We estimated that in vitro assays underestimated in vivo potency by 6- to 7-fold against Omicron variants. Our model suggested that because polymerase chain reaction detects molnupiravir mutated variants, the true reduction in non-mutated viral RNA was underestimated by approximately 0.4 log_10_ in the two trials conducted while Omicron variants dominated. Viral area under the curve estimates differed significantly between non-mutated and mutated viral RNA. Our results reinforce past work suggesting that in vitro assays are unreliable for estimating in vivo antiviral drug potency and suggest that virologic endpoints for respiratory virus clinical trials should be catered to the drug mechanism of action.

## Abstract 2

We use mathematical models to synthesize results of three trials of molnupiravir against SARS-CoV-2 to demonstrate that the drug has greater potency against omicron variant than prior variants. In addition, its potency against omicron is substantially underestimated by standard PCR assays.

## Introduction

Molnupiravir is an antiviral prodrug that induces errors in the SARS-CoV-2 genome, which typically renders the virus unable to replicate further (1). In the randomized double-blinded MOVe-OUT trial, which enrolled unvaccinated individuals when Delta, Mu, and Gamma variants of concern (VOCs) were circulating, molnupiravir reduced hospitalization by 50% and viral load after treatment (day 5) by 0.3 log_10_ relative to placebo (2). In the adaptive platform PANORAMIC trial, which enrolled vaccinated individuals when Omicron VOCs were circulating, hospitalization rates were only 1% in both arms, but molnupiravir lowered viral load after treatment by 0.94 log_10_ relative to usual care (3). In the adaptive platform PLATCOV trial, which enrolled low-risk individuals when Omicron VOCs were circulating, molnupiravir lowered viral load after treatment by 1.09 log_10_ relative to usual care (4). Taken together, these trials demonstrate that molnupiravir has both clinical and virologic efficacy that varied across trials and viral variants.

Overall, use of molnupiravir has been lower than that of nirmatrelvir/ritonavir based on lower reduction in hospitalization in MOVe-OUT relative to the EPIC-HR trial for nirmatrelvir/ritonavir (5). A concern has also been raised that molnupiravir’s mechanism of action could generate novel mutants that persist after cessation of treatment (6), and then spread in the population (7). Nevertheless, the PANORAMIC and PLATCOV trial results suggest high potency, and molnupiravir is still considered in individuals in whom nirmatrelvir/ritonavir is contraindicated and in combination with other drugs in immunocompromised hosts (8, 9). There is currently no explanation for the disparate antiviral effects in MOVe-OUT versus PANORAMIC and PLATCOV. Moreover, the fact that polymerase chain reaction (PCR) detects drug-altered viral RNA molecules (6) has not been considered in the analysis of trial outcomes. A study in ferrets highlights the potential importance of this effect; while molnupiravir and nirmatrelvir/ritonavir dramatically lowered levels of infectious SARA-CoV-2 titers, only nirmatrelvir/ritonavir lowered total viral RNA levels (10). Possible discrepancies between PCR detection and reduction in infectious virus have been repeatedly recognized for this class of drugs (11–13).

We previously used clinical trial simulation to reproduce results from nirmatrelvir/ritonavir trials for SARS-CoV-2 (14, 15). We first validated a viral immune dynamic model (VID) against a very large prospective cohort of infections that included multiple VOCs (15). We used sets of model parameters that reflect diverse virologic output to create simulated cohorts for the control arms of trials. We then integrated pharmacokinetic (PK) and pharmacodynamic (PD) models for nirmatrelvir/ritonavir with the VID models to simulate treatment arms (14). This approach recapitulated mean viral load reduction in the EPIC-HR and PLATCOV trials, as well as individual viral load trajectories in PLATCOV. The validated model was then used to explain the high frequency of virologic and concurrent symptomatic rebound following use in the community (16), despite very low levels of virologic and symptomatic rebound in the EPIC-HR trial (17, 18). Model output suggests that extending therapy from 5 to 10 days would nearly eliminate rebound (14), a result confirmed with modeling of separate data (19).

Here we expand this approach to develop a new joint VID-PKPD model to account for the unique mechanism of action of molnupiravir. We fit the model to results from the MOVe-OUT, PLATCOV, and PANORAMIC trials. Our results suggest that quantitative viral PCR likely underestimates the reduction in non-mutated viral RNA and therefore the true potency of molnupiravir during Omicron infections.

## Results

### Overview.

We established and validated our clinical trial simulation platform in multiple steps. (a) We previously developed and validated a SARS-CoV-2 VID model by fitting competing models to viral load trajectories from participants in a large natural history study of infected and untreated individuals (15, 20). (b) We established and validated a PK model for molnupiravir by fitting competing models to serial plasma drug concentrations from a phase I clinical trial. (c) We estimated dose-response model parameters by fitting a PD model to in vitro dose escalation data. (d) We merged the VID, PK, and PD models into a single mathematical model to allow fits to viral load data from clinical trials. (e) We fit the combined model to mean viral load reduction in the treatment versus control arms of the MOVe-OUT, PLATCOV, and PANORAMIC trials. (f) We fit the combined models to individual viral load trajectories from treated and untreated participants in the PLATCOV and PANORAMIC trials. (g) We estimated the potency adjustment factor (paf) for molnupiravir in each trial; the paf is the ratio of molnupiravir’s in vitro EC_50_ (estimated in step c) to its in vivo EC_50_ (estimated in steps e and f); the in vivo EC_50_ is defined as the plasma drug concentration associated with 50% reduction in viral replication. (h) We used the estimated pafs to quantify a relevant value for drug developers and clinical trialists: the average in vivo potency of the drug throughout the treatment course. (i) We further validated the model by performing counterfactual simulations, which assume treatment for participants in the control arm and usual care for participants in the treatment arm, and then comparing these simulations to trial data. (j) We used the validated model to perform detailed analysis of each trial’s outcomes and to assess virologic endpoints that account for the mutagenic mechanism of action of molnupiravir. (k) We used the validated model to simulate different doses and dosing strategies for optimization purposes.

### Viral immune dynamic, PK, and PD clinical trial simulation models.

**Figure 1:**
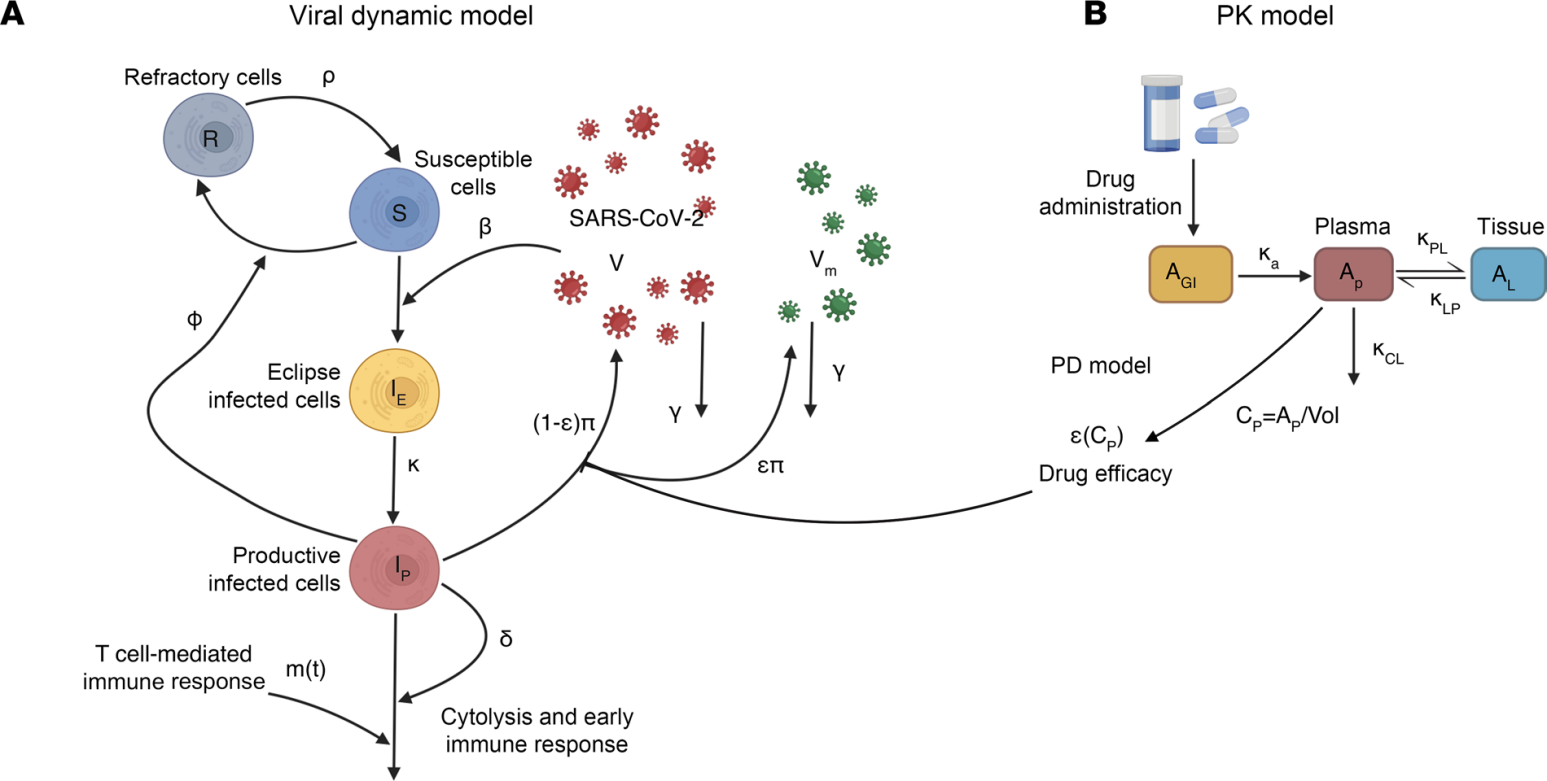
Schematic of the viral dynamic model and molnupiravir PKPD model. (**A**) In the viral dynamic model, S represents the susceptible cells, *I_E_* is the eclipse infected cells, *I*_p_ is the productively infected cells, V is the non-mutated viruses, and V_m_ is the viruses mutated by treatment. The productively infected cells are cleared by early and late T cell–mediated immune response at rates *δ* and *m*(*t*). *β* is the infectivity rate, *Φ* is the rate of conversion of susceptible cells to refractory cells, and *ρ* is the rate of reversion of the refractory cells to susceptible cells. Productively infected cells produce viruses at the rate *π*, and free viruses are cleared at the rate *γ*. (**B**) Two-compartmental PK model with oral administration of the drug which models the amounts of the drug in gut tissue (A_GI_), plasma (A_P_), and the respiratory tract (A_L_). *κ_a_* is the rate of absorption of the drug from gut to plasma, *κ_PL_* and *κ_LP_* are the rates of transfer of the drug from plasma to the respiratory tract and back, *κ_CL_* and is the rate at which the drug clears from the body. Vol is the estimated plasma volume and *C_p_* is the concentration of the drug in plasma. *ε*(*C_p_*) is the efficacy of the drug in converting produced viruses into mutated, non-infectious viruses. Created in BioRender. https://BioRender.com/zm3u454.

We previously described our VID model that was fit to diverse serial viral loads from 1510 SARS-CoV-2 infected individuals in the National Basketball Association (NBA) cohort (15, 20). The model assumes a finite number of susceptible cells and an eclipse phase delays viral production by infected cells. In keeping with an early innate immune response, susceptible cells become refractory to infection in the presence of infected cells but also revert to a susceptible state at a constant rate. Infected cells are cleared by cytolysis and delayed acquired immunity, which is activated in a time-dependent fashion (Figure 1A). We used a mixed-effect population approach implemented in Monolix to estimate model parameters (21).

To reproduce levels of molnupiravir, we used a 2-compartment PK model (Figure 1B). Using Monolix and the mixed-effect population approach, we estimated parameter values by fitting the model to the average plasma concentration of healthy individuals (22). The model closely recapitulated observed drug levels following multiple doses of 50, 100, 200, 300, 400, 600, and 800 mg given twice daily for 5 days (Supplemental Figure 1 and Supplemental Table 1; supplemental material available online with this article; https://doi.org/10.1172/JCI192052DS1); 800 mg twice daily for 5 days is the clinically recommended dose that was assessed in MOVe-OUT, PLATCOV, and PANORAMIC. The estimated value for the transition rate from plasma to peripheral compartment (*κ_PL_*) was dose-dependent in the form of *κ_PL_* = *κ*_PL,1_*Dose^α^*, with decreasing *κ_PL_* as the dose increases. While there are no tissue PK data available to explain the decreasing transition rate from plasma to the peripheral compartment estimated by our model, we hypothesize that this effect might be due to saturation of tissue uptake at higher concentrations or a shift in the balance of bidirectional transport between plasma and peripheral compartments at higher concentrations. All other PK parameters were dose independent.

For the PD model, we assumed drug efficacy follows a Hill equation with respect to concentration using data from our own lab. We parameterized the model using in vitro efficacy data collected at different concentrations (details in Methods, Supplemental Figure 2A and Supplemental Table 2) (23). Our estimates for in vitro EC_50_ approximated values from other groups, which differed according to VOC and laboratory (Supplemental Figure 2B) (24, 25).

We combined the VID, PK, and PD models by using treatment efficacy to convert non-mutated virus to mutated virus, both of which are assumed to be detected with polymerase PCR assays, given the low probability of drug-induced mutations in the PCR primer region. A similar mechanism of action has been used in modeling ribavirin treatment against the hepatitis C virus and HIV protease inhibitors (26, 27). For molnupiravir, this assumption is based on the observed drug-induced mutation rate of approximately 1 mutation per 2000 base pairs (28). Given the average length of most PCR primers of approximately 25 base pairs, the chance of the primer remaining unmutated after treatment is (1999/2000)^25^, or 98.76%.

**Figure 2:**
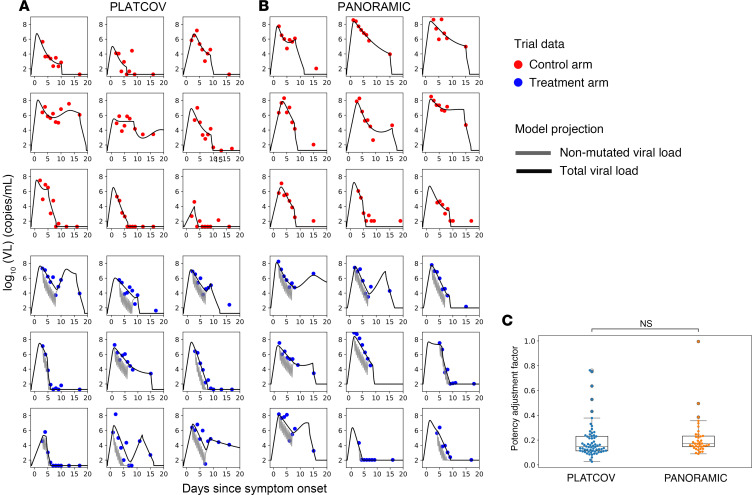
Mathematical model fits of SARS-CoV-2 viral load over time to a subset of study participants in PLATCOV and PANORAMIC receiving no treatment (control) or molnupiravir. (**A**) Model fits to 9 control and 9 treatment participants in PLATCOV. (**B**) Model fits to 9 control and 9 treatment participants in PANORAMIC. Remaining model fits are in the supplementary materials. (**C**) Individual estimates for potency adjustment factor (in vivo EC_50_/in vitro EC_50_ ratio) in the 2 trials (center line, median; box limits, upper and lower quartiles; whiskers, 1.5× interquartile range). The statistical comparison was performed using Mann-Whitney *U* test.

A limitation of viral load data from the included clinical trials is that it lacks early presymptomatic measurements to estimate the viral expansion slope. To further train the model, we included untreated Omicron-infected participants from the NBA cohort (*n* = 1023) in the fitting population to inform rates of viral upslope in the trials (15). We first fit the combined model to individual viral load data from 149 low-risk, symptomatic vaccinated participants infected with Omicron VOCs in the PLATCOV trial (65 treated and 84 controls) and from 80 high-risk, symptomatic vaccinated participants infected with Omicron VOCs in the PANORAMIC trial (38 treated and 42 controls) (Figure 2, A and B, and Supplemental Figures 3–6).

**Figure 3:**
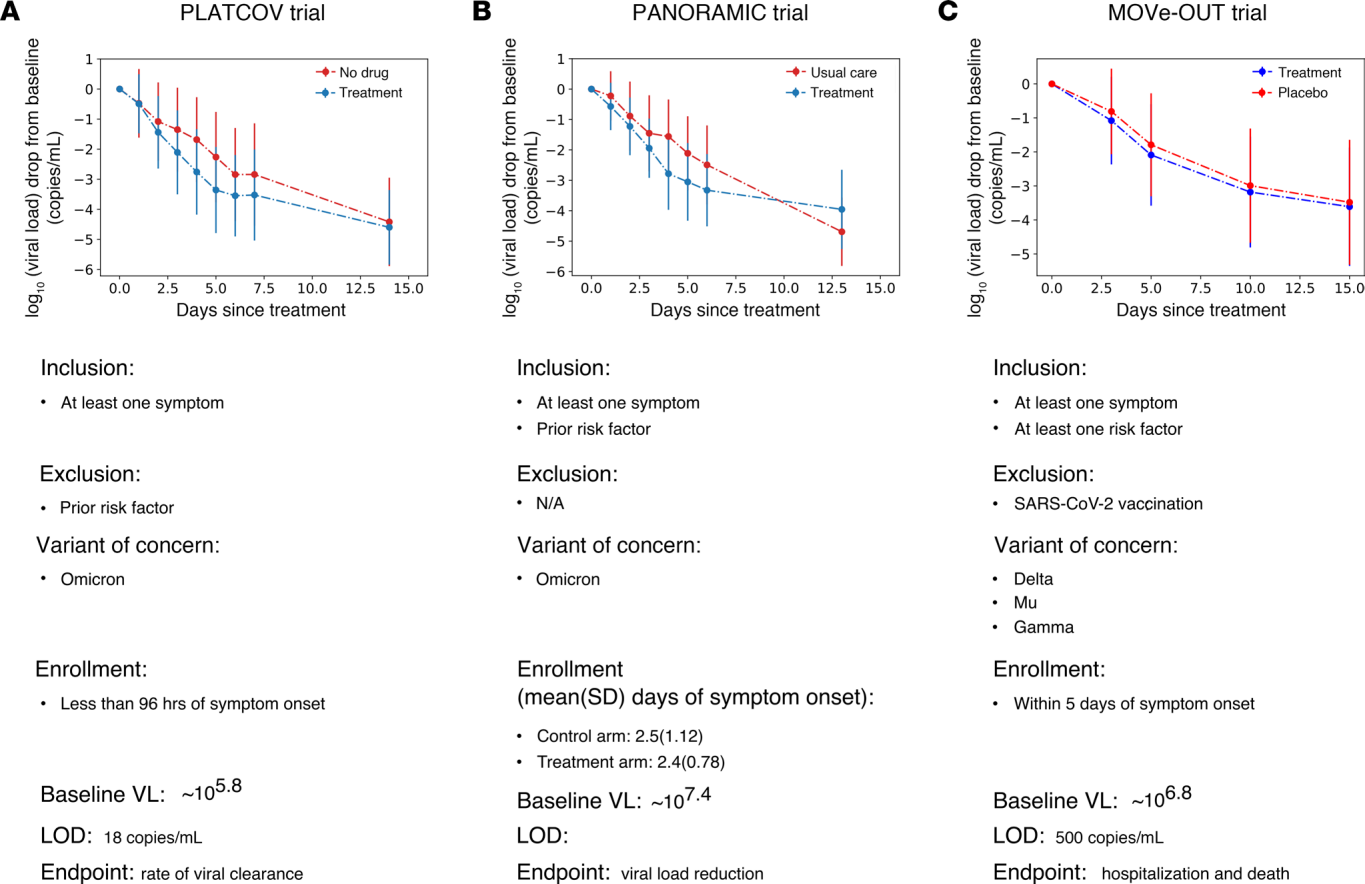
Mean viral load reduction in the 3 trials that are targets for model fitting. (**A**) PLATCOV included individuals with no risk factor for severe disease infected by the Omicron variant, and enrolled within 96 hours of symptom onset. (**B**) PANORAMIC included individuals with at least one risk factor for severe disease infected by the Omicron variant, and enrolled on average 2.5 days since symptom onset. (**C**) MOVe-OUT included high-risk, unvaccinated individuals, infected by Delta, Mu, and Gamma variants, and enrolled within 5 days of symptom onset. Trials also differed according to swabbing site, viral PCR assay, enrollment viral load, and lower limit of detection.

**Figure 4:**
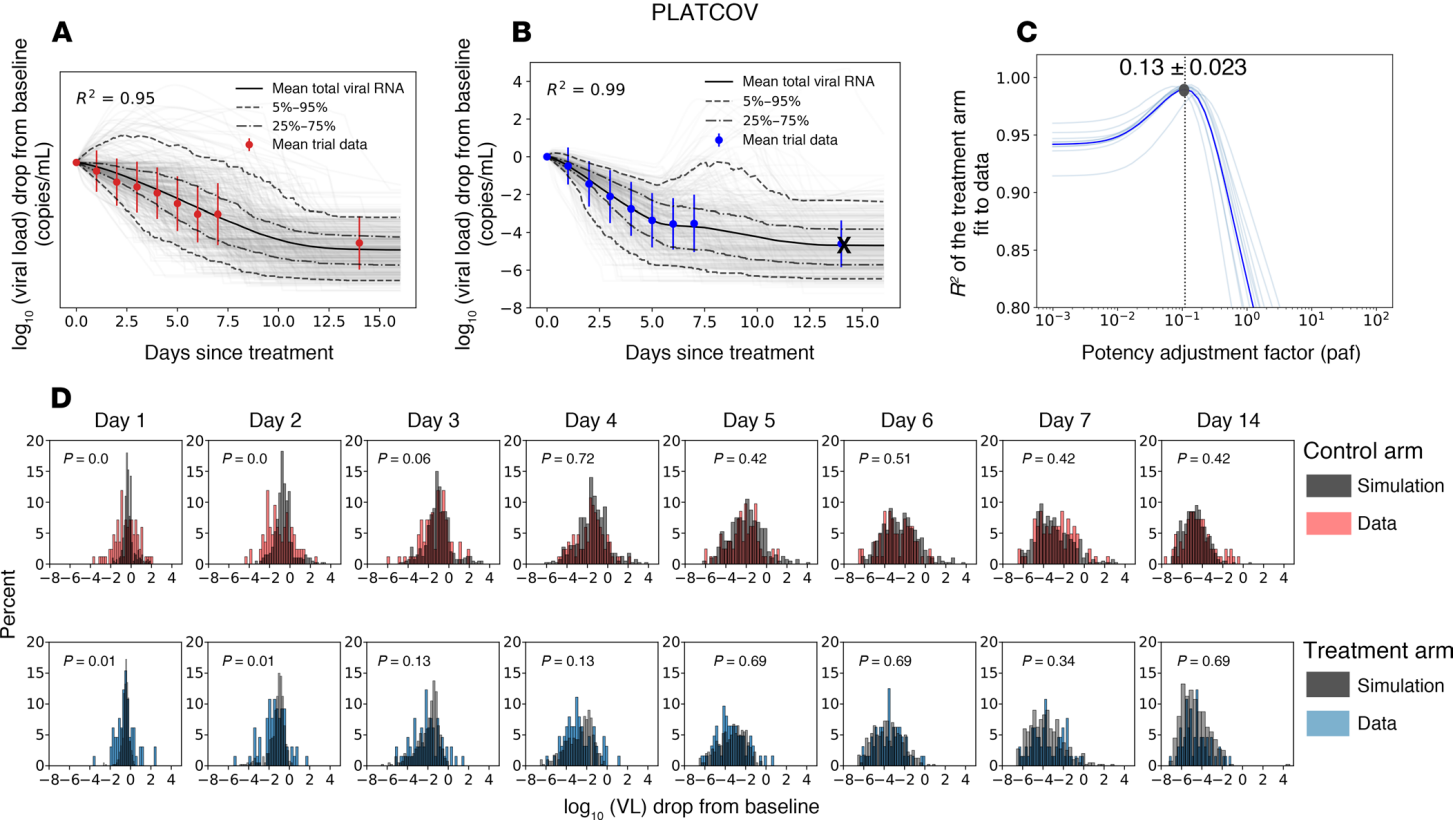
Model fit to virologic trial outcomes for PLATCOV. Results include the fit to drop in viral load from baseline for (**A**) control groups and (**B**) treatment groups. Control arm data are shown in red, treatment arm data in blue, gray lines are the simulated viral load drop for each individual, and solid lines are the mean viral load drop. (**D**) Comparing individual variability of data versus simulation in control and treatment arms. The 2-sided Kolmogorov-Smirnov test was used to compare the distributions. Adjusted *P* values were calculated using the Benjamini-Hochberg method and represent dissimilarity between observed and simulated distributions. (**C**) Estimates for the potency adjustment factor (paf). To only capture the effect of treatment and address potential identifiability issues, data from the first 7 days after baseline were used to estimate the paf. Therefore, the crossed-out data points were not included in the calculation of *R*^2^.

**Figure 5:**
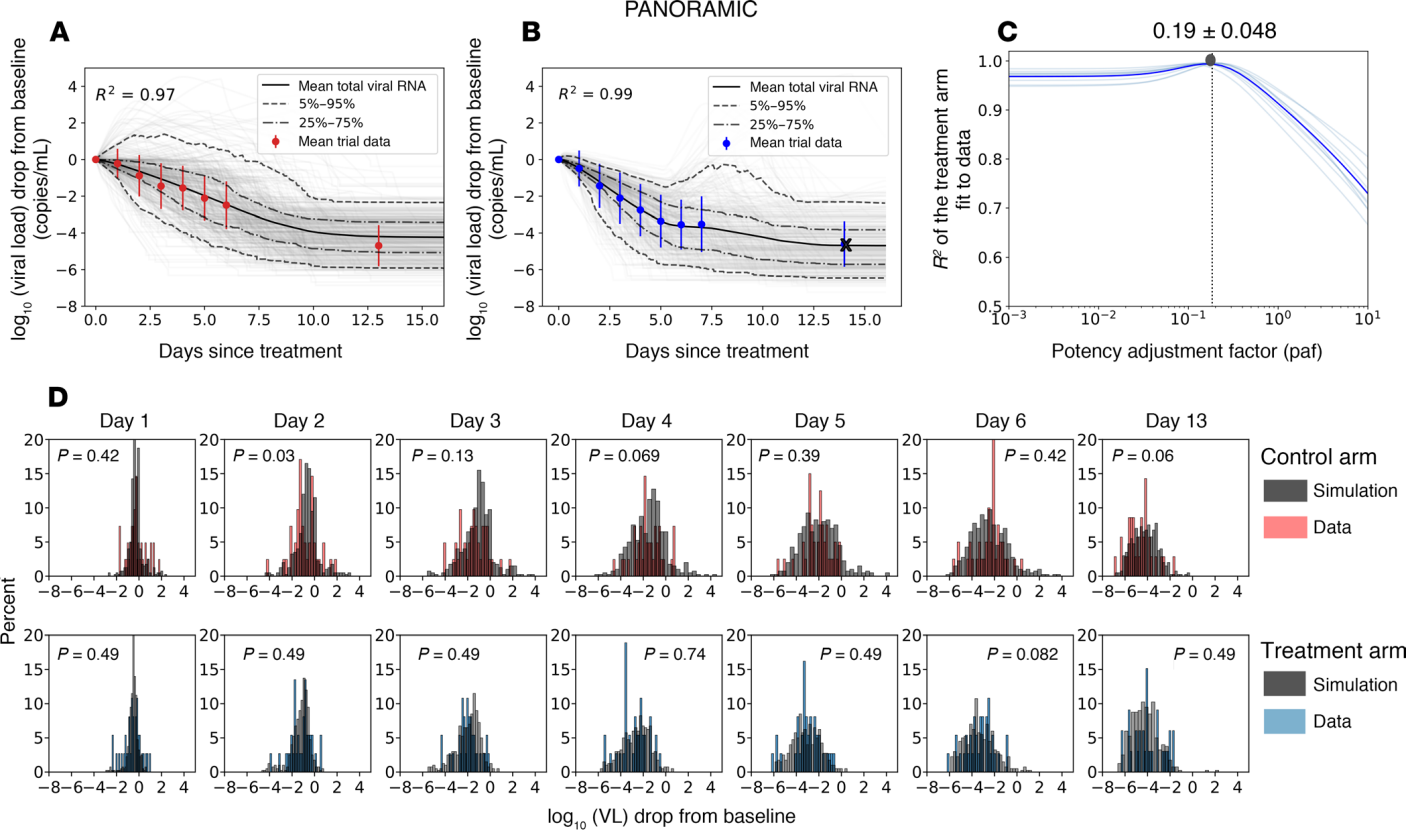
Model fit to virologic trial outcomes for PANORAMIC. Results include the fit to drop in viral load from baseline for (**A**) control groups and (**B**) treatment groups. Control arm data are shown in red, treatment arm data in blue, gray lines are the simulated viral load drop for each individual, and solid lines are the mean viral load drop. (**D**) Comparing individual variability of data versus simulation in control and treatment arms. The 2-sided Kolmogorov-Smirnov test was used to compare the distributions. Adjusted *P* values were calculated using the Benjamini-Hochberg method and represent dissimilarity between observed and simulated distributions. (**C**) Estimates for the potency adjustment factor (paf). To only capture the effect of treatment and address potential identifiability issues, data from the first 7 days after baseline were used to estimate the paf. Therefore, the crossed-out data points were not included in the calculation of *R*^2^.

**Figure 6:**
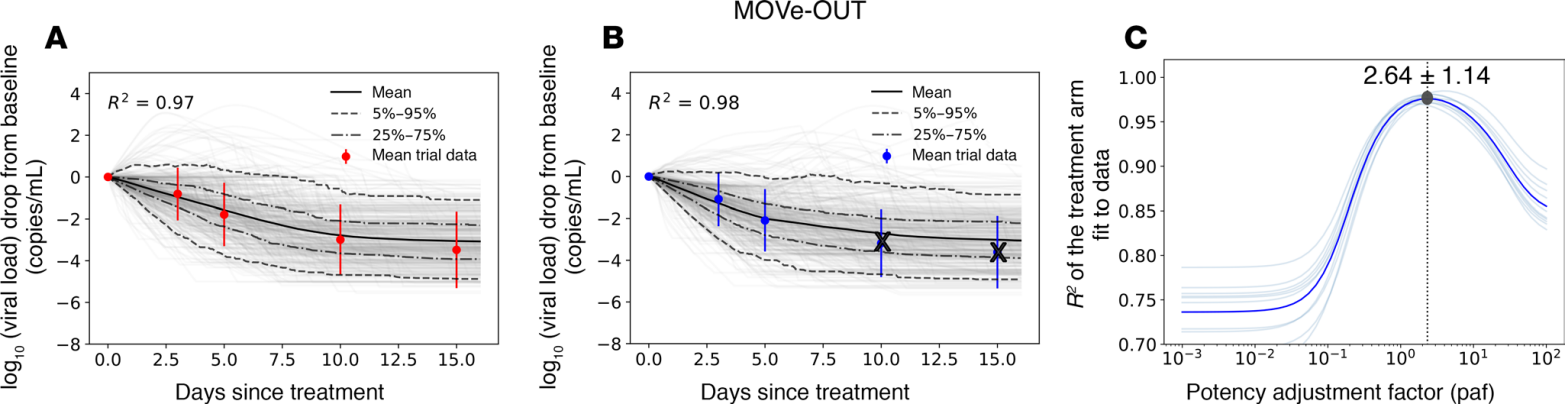
Model fit to virologic trial outcomes for MOVe-OUT. (**A**) Control groups and (**B**) treatment groups. Control arm data are shown in red, treatment arm data in blue, gray lines are the simulated viral load drop for each individual, and solid lines are the mean viral load drop. (**C**) Estimates for the potency adjustment factor (paf). To only capture the effect of treatment and address potential identifiability issues, data from the first 7 days after baseline were used to estimate the paf. Therefore, the crossed-out data points were not included in the calculation of *R*^2^.

We next fit the combined model to trial endpoint data (mean viral load drop from baseline) reported in 3 randomized, controlled trials: PLATCOV (Figure 3A and Figure 4, A–D) (4), PANORAMIC (Figure 3B and Figure 5, A–C) (3), and the MOVe-OUT trial with 1093 high-risk unvaccinated symptomatic individuals infected with pre-Omicron VOCs (549 treated + 544 placebo, Figure 3C and Figure 6, A–C) (2). All model fitting was performed using Monolix with non-linear mixed-effect approaches described in the Methods.

### Model fitting to individual viral load trajectories in PLATCOV and PANORAMIC.

For each participant, we defined the in vivo EC_50_ as the plasma drug concentration required to inhibit viral replication by 50% and the paf as the ratio between the in vivo and in vitro EC_50_ (29, 30). To estimate the paf for each participant, we fit the combined VID-PKPD model to individual viral load data from both arms of the PLATCOV and PANORAMIC trials, as well as Omicron-infected individuals in the NBA cohort. We achieved good model fit to individual viral load trajectories in the control and treatment arms of PLATCOV (Figure 2A and Supplemental Figures 3 and 4) and PANORAMIC (Figure 2B and Supplemental Figures 5 and 6). The model projected higher levels of total detected SARS-CoV-2 RNA in most participants relative to non-mutated viral RNA (Figure 2, A and B). We estimated a range of individual paf values with similar mean and median values estimated for both trials (Figure 2C). These values suggest that in vivo potency of molnupiravir is on average 6- to 7-fold higher than estimates based from our in vitro data. Each participant had an estimated paf less than 1, indicating that enhanced potency in vivo is necessary to accurately model the data.

### Model fit to trial virologic endpoint data from PLATCOV, PANORAMIC, and MOVe-OUT.

As a second approach, we assessed whether a virtual cohort strategy where control participants are modeled using estimated parameter values from preexisting cohorts can predict virologic trial endpoints. This approach is necessary for situations where individual viral load data are not publicly available as with MOVe-OUT and demonstrates that the model can reproduce the primary virologic endpoint of each study. We simulated virtual cohorts using the combined VID-PKPD model and fit results to viral load decay from baseline in the 3 trials. For each trial arm, we randomly selected 400 individuals from the NBA cohort with the closest matching viral variant, symptom, and vaccine status and used their estimated individual viral dynamic parameters in simulations. To address variability in timing of baseline viral load measurement relative to infection, we randomly assigned all individuals an incubation period selected from a variant-specific gamma distribution found in the literature (31, 32). Treatment start day was randomly selected from a distribution based on observed enrollment windows in the 3 trials. Due to the lack of individual PK data, the same estimated population PK parameters were used for all simulated treated individuals (Supplemental Table 1). PD parameters were randomly selected from a log-normal distribution with estimated mean and standard error from in vitro assay results (Supplemental Table 2).

Our model closely reproduced kinetics of viral decay in the PLATCOV control (Figure 3A and Figure 4A) and treatment arms (Figure 3A and Figure 4B) and estimated a paf of 0.13 (Figure 4C), similar to our median estimate using individual fits (Figure 2C). The model also predicted individual-level variability in virologic responses observed in PLATCOV, including instances of increased viral load following therapy (Figure 4D). We compared simulated and actual distributions of viral load change among trial participants in the control and treatment arms. On most posttreatment days, simulated and actual distributions were not statistically dissimilar. Wider distributions of observed versus simulated viral load change were noted on postrandomization days 1 and 2 for control and treatment (Figure 4D), likely due to noise in viral load data from oral swabs: differences of 1–2 logs were often noted between replicates collected from PLATCOV participants at equivalent time points, particularly on days 1 and 2 (14).

Similarly, our model closely reproduced kinetics of viral decay in PANORAMIC in control (Figure 3B and Figure 5A) and treatment arms (Figure 3B and Figure 5B) and estimated a paf of 0.19 (Figure 5C), similar to our median estimate using individual fits (Figure 2C). The model also predicted individual-level variability in virologic responses observed in PANORAMIC, including instances of increased viral load following therapy (Figure 5D). We compared simulated and actual distributions of viral load change among trial participants in the control and treatment arms. On all posttreatment days other than day 2, control, simulated, and actual distributions were not statistically dissimilar (Figure 5D). This likely indicates less noise in viral load data from nasopharyngeal swabs collected in PANORAMIC relative to oral swabs in PLATCOV.

Finally, the model reproduced kinetics of viral decay in MOVe-OUT in control (Figure 6A) and treatment arms (Figure 6B) but estimated a higher paf of 2.64 (Figure 6C). The higher paf maps to the far less substantial viral load reduction in MOVe-OUT relative to the other 2 trials, which in turn might be explained by less potency against pre-Omicron variants that has been observed experimentally (33).

As a further validation step, we performed counterfactual simulations by presuming treatment (800 mg twice daily for 5 days) for the participants in the control arms (treatment counterfactual) and assuming no treatment for participants in the treatment arm (control counterfactual). Counterfactual control simulations slightly overestimated late viral loads for PLATCOV (Supplemental Figure 7A) and PANORAMIC (Supplemental Figure 8A). This may be because therapy suppresses acquired immune responses, which are not captured in our model (19). Counterfactual treatment simulations fit the data well for PLATCOV (Supplemental Figure 7B) and PANORAMIC (Supplemental Figure 8B). Simulations occasionally predicted viral rebound following treatment (Supplemental Figure 7, C and D, and Supplemental Figure 8, C and D).

### Estimates of reduction in fully mutated viruses versus non-mutated SARS-CoV-2 RNA.

**Figure 7:**
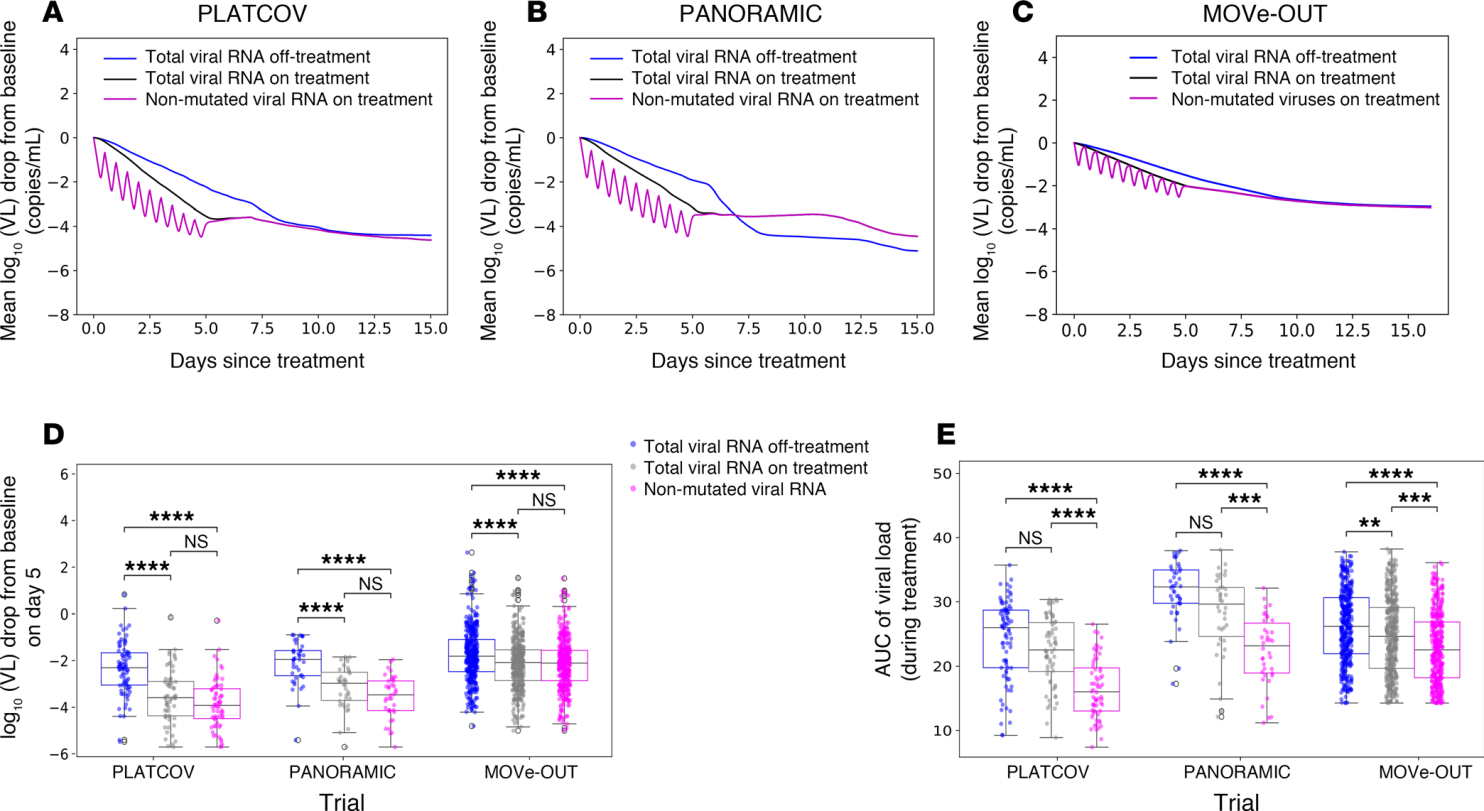
PCR underestimates the true reduction in non-mutated SARS-CoV-2 RNA in PLATCOV and PANORAMIC. Simulated mean viral loads including non-mutated viral RNA in (**A**) PLATCOV, (**B**) PANORAMIC, and (**C**) MOVe-OUT. (**D**) Individual viral load reduction at day 5 in the simulated control group (blue), simulated treatment group total viral RNA (gray), and simulated treatment group non-mutated viral RNA (pink) in the 3 trials, showing no statistical difference between total and non-mutated viral RNA despite a lower median. (**E**) Individual viral AUC from the start of the treatment through day 5 in the simulated control group (blue), simulated treatment group total viral RNA (gray), and simulated treatment group non-mutated viral RNA (pink), showing a statistical difference between total and non-mutated viral RNA in all 3 trials. (**D** and **E**) Box-and-whisker plots include the interquartile range (IQR) with whiskers equaling 1.5 times the IQR.

We used our optimized model with solved paf to project the trajectory of non-mutated viral RNA during treatment relative to values measured with PCR, which detects viral RNA with drug-induced mutations (6). In PLATCOV (Figure 7, A and D, and Table 1) and PANORAMIC (Figure 7, B and D, and Table 1), owing to higher drug potency, total SARS-CoV-2 viral RNA on treatment exceeded non-mutated viral RNA by approximately 0.37 and 0.44 log_10_, respectively, on day 5, suggesting that measured endpoints underestimate the drug’s true antiviral effect. However, these differences did not achieve statistical significance, perhaps because estimated total and non-mutated viral RNA levels converged at drug trough. In MOVe-OUT (Figure 7, C and D, and Table 1), there was no significant difference between total SARS-CoV-2 viral RNA on treatment and non-mutated viral RNA.

In all 3 trials, the models suggest that non-mutated viral loads during treatment may be lowest at drug peak, reflecting the short plasma half-life of molnupiravir. Therefore, the cumulative effect of drug is best estimated with viral area under the curve (AUC), which accounts for highly variable drug activity over time due to short drug half-life. By this estimate, the reduction in non-mutated viral RNA far exceeded that of total measured viral RNA in all 3 trials (Figure 7E). In the case of MOVe-OUT, this may explain why significant clinical benefit was associated with only a marginal reduction in observed decline in total viral RNA. This also suggests that there might be utility to measure viral load after drug peak and trough for agents with short half-life as viral loads may differ by a full order of magnitude according to drug level.

To mimic endpoints in PLATCOV, we estimated the slope of viral decay of the total viral RNA on and off treatment as well as the non-mutated viral RNA by fitting a linear model to the simulated log_10_(viral load) data on days 0, 1, 2, 3, 4 and 5 after the start of the treatment. The median clearance half-life (*t*_1/2_ = log_10_[0.5]/slope) in our simulations of PLATCOV control and treatment arms agreed with the clearance half-life observed in the trial (Supplemental Table 3). Our results showed small differences (non-significant) between the clearance rates for the total versus the non-mutated viral RNA in the treated groups in PLATCOV and PANORAMIC (Supplemental Figure 9).

### Differences in trial participants and model parameters.

We next compared features of each trial as they related to model predictions by assessing the viral dynamic range (Supplemental Figure 10A). Control participants in PLATCOV had lower mean viral loads throughout the course of infection relative to PANORAMIC and MOVe-OUT (Supplemental Figure 10B). Given that PLATCOV and PANORAMIC enrolled participants with the Omicron variant, we surmise that these differences relate to demographic differences in study participant, slightly shorter estimated time to treatment in PANORAMIC versus PLATCOV estimated by our model (*t*_0_ in Supplemental Figure 11), sampling site, and/or characteristics of the PCR assay used in the studies. The trials also employed different limits of detection which impacted observed reductions in viral load (Supplemental Figure 10B). Model parameters were largely equivalent between studies and between treatment and control arms, reflecting the flexibility of the model (Supplemental Figure 11). The parameter governing transition of susceptible cells to a refractory state was higher in PLATCOV relative to PANORAMIC which likely was necessary to achieve lower viral loads overall and may reflect the younger study participants or different immune dynamics in the oral versus nasal compartment (Supplemental Figure 11).

### Antiviral potency, viral load assessment, and trial design impact observed antiviral reduction.

**Figure 8:**
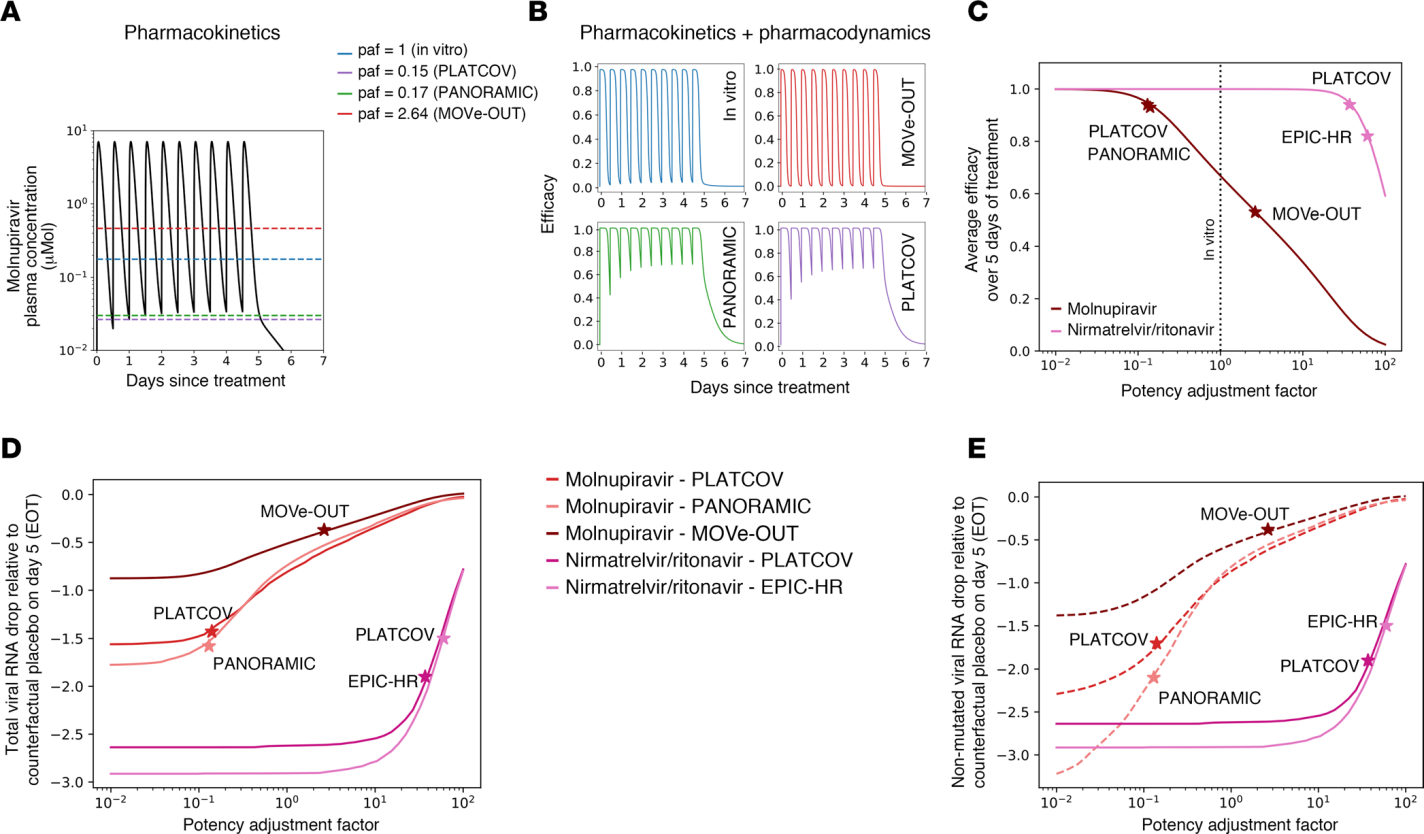
Relationship of drug pharmacokinetics and pharmacodynamics to in vivo potency and viral load reduction, comparing trial design. (**A**) Molnupiravir plasma concentration during 5 days of treatment with 800 mg molnupirvir given twice daily. The dashed lines mark the EC_50_ with different paf values that differ by trial. For paf = 0.13, drug levels are almost entirely above the EC_50_. (**B**) Dynamic shifts in molnupiravir efficacy for different paf values that differ by trial. Efficacy only drops minimally at trough levels when paf is low (i.e., 0.14 and 0.13 in PLATCOV and PANORAMIC) but drops significantly at trough levels in MOVe-OUT. (**C**) Drug potency of SARS-CoV-2 antivirals according to trial. The in vivo efficacy of molnupiravir in PLATCOV and PANORAMIC trials is close to the in vivo efficacy of nirmatrelvir/ritonavir in the PLATCOV trial and higher than EPIC-HR. MOVe-OUT potency is significantly lower due to a higher paf and higher in vivo EC_50_ value. (**D**) Simulated mean drops in total viral RNA from baseline relative to untreated arms on day 5 in the 3 molnupiravir trials and 2 nirmatrelvir/ritonavir trials. (**E**) Simulated mean drops in non-mutated viral RNA from baseline relative to counterfactual placebo on day 5 in the 3 molnupiravir trials and 2 nirmatrelvir/ritonavir trials. In the molnupiravir trials, total viral RNA drops less than non-mutated viral RNA due to PCR detection of drug-mutated viral RNA. Total possible reduction in non-mutated SARS-CoV-2 RNA is less for MOVe-OUT than PLATCOV and PANORAMIC due to higher initial viral loads and lower values of detection in the trials.

We next combined PK and PD models to assess the average efficacy of the drug during days 0–5 in all 3 trials (Figure 8, A and B). Due to the short half-life of the drug, its plasma concentrations fell below the therapeutic levels (in vivo EC_50_) in MOVe-OUT trial (red dashed line in Figure 8A), while remaining above the in vivo EC_50_ in PLATCOV and PANORAMIC (purple and green dashed lines in Figure 8A). We then calculated the average efficacy (see Equation 3 in Methods) assuming different in vivo potencies (i.e., different paf) and noted an efficacy of 53% in MOVe-OUT (Figure 8C). The efficacy of molnupiravir in PLATCOV (94%) and PANORAMIC (95%) was similar to that of nirmatrelvir in PLATCOV (94%) and EPIC-HR (82%), owing to a much lower paf for molnupiravir relative to nirmatrelvir/ritonavir (Figure 8C).

We simulated each trial with its specific design (timing of treatment and limit of detection [LOD]), assuming different paf values (Figure 8, D and E). These potencies are mapped to different reductions in viral load relative to placebo/usual care. Total viral RNA reduction in PANORAMIC and PLATCOV exceeded that in MOVe-OUT, owing to lower paf (Figure 8D), but also due to a larger viral dynamic range (defined as the distance from baseline viral load to the lower threshold PCR (LOD) (Supplemental Figure 10B), which allows for a greater observed reduction in viral load. PANORAMIC used an LOD of 109 (imputed as 50 copies/mL), and PLATCOV used an LOD of approximately 18 copies/mL, but MOVe-OUT had a higher LOD of 500 copies/mL. PANORAMIC also had much higher average starting viral loads (7.4 log_10_[copies/mL]) versus PLATCOV (5.8 log_10_[copies/mL]) and MOVe-OUT (6.8 log_10_[copies/mL]) (Supplemental Figure 10B). Molnupiravir approached maximal possible total viral RNA reduction in PANORAMIC and PLATCOV, whereas protease inhibitors could still achieve greater viral load reduction at lower paf (Figure 8D), as recently observed with ensitrelvir (34). Overall, assays that maximize viral load detection and have lower limits of detection can detect greater virologic reduction in trials.

The greater possible reduction in total viral load for protease inhibitors relative to mutagenic drugs like molnupiravir owes to different mechanisms of action. Model projected reductions in non-mutated viral RNA reduction in PANORAMIC and PLATCOV approximated viral load reductions observed in EPIC-HR and PLATCOV on nirmatrelvir/ritonavir (Figure 8E). This suggests that PCR detection of mutated viruses underestimates true molnupiravir potency, and that a more potent mutagenic agent could accrue further virologic benefit even if the total reduction in viral RNA does not increase.

### Optimization of molnupiravir therapy to avoid viral rebound.

Instances of viral rebound were observed in PLATCOV and PANORAMIC and have been observed following molnupiravir treatment (4, 35). We analyzed higher doses and prolonged therapy and noted that, as with nirmatrelvir (14), prolonging therapy is a better method to prevent rebound than increasing dose (Supplemental Figure 12). While the calculated probability of rebound depends on the rebound definition (see Methods), we showed previously that the general trend as a function of different changes in the treatment regimen will hold (14). In addition, due to the limited follow-up window of the trials, our model may predict instances of rebound that occur outside the window of observed data.

## Discussion

We recapitulated the virologic results of 3 clinical trials for molnupiravir with our combined clinical trial simulation VID-PKPD models. Model output highlights key differences in viral load reduction between the trials and identifies mechanisms to explain these discrepancies. The MOVe-OUT trial was associated with significantly less reduction in viral load between treatment and control arms than the other 2 trials. Accordingly, the average drug efficacy over the dosing interval (53%) was lower in this trial, and our estimate for paf was 2.64, signifying marginally lower potency in vivo than in vitro. This result is compatible with 0.4-fold lower median EC_50_ values for molnupiravir in vitro against Omicron relative to prior variants (33), although explanations other than viral variant cannot be ruled out. The higher paf in MOVe-OUT permitted drug troughs below the EC_50_, limiting potency throughout the dosing interval. In PLATCOV and PANORAMIC, our model suggests drug levels remain above the in vivo EC_50_ throughout the dosing interval, although potency does fluctuate according to drug level.

The projected drug efficacy in PLATCOV and PANORAMIC was considerably higher (94% and 95%, respectively), and the paf was estimated to be 0.14 and 0.13, indicating greater potency in vivo than in vitro. We have applied our clinical trial simulation technique to multiple drugs for SARS-CoV-2 (14), HSV-2 (29, 30), and HIV (36), and this is the first time we have identified this trend. Allowing molnupiravir to be more potent in vivo in our modeling of PLATCOV and PANORAMIC was necessary to capture the much greater reduction in total viral RNA relative to off-treatment in these studies (1.09 log_10_ and 0.94 log_10_, respectively, versus 0.3 log_10_ in MOVe-OUT). Another modeling study was also unable to recapitulate viral load reductions in PANORAMIC when assuming equivalent in vitro and in vivo EC_50_ levels (37). Instead of adjusting the in vivo EC_50_ as we did, the authors assumed a secondary drug mechanism of action, reduction in viral replication rate. However, to our knowledge, viral mutagenesis is the only experimentally proven mechanism of action of molnupiravir (38–40). Another study reinforced the primacy of the mutagenic mechanism of action by showing very high drug-induced nucleotide substitution rates after 2 days of treatment (41).

A key outcome of our analysis is the prediction that SARS-CoV-2 PCR likely underestimates molnupiravir potency because it detects drug-mutated viral RNA (6). This appears to be most significant when antiviral potency is higher, as in PANORAMIC and PLATCOV, leading to a 0.48–0.59 log_10_ underestimation of reduction in non-mutated virus. Our results suggest that use of standard PCR for assessing SARS-CoV-2 levels may lead to underestimation of drug potency. Multiprobe assays, as have been used for the HIV reservoir, may improve specificity for viruses that remain intact and replication competent (42). Viral culture is potentially a useful metric but lacks sufficient sensitivity and precision and is too labor intensive to serve as a viable trial endpoint (16). In a ferret model, molnupiravir lowered levels of culturable virus while not lowering total viral RNA levels relative to control (10). In PANORAMIC, molnupiravir did not impact culture detection for virus between days 2 and 5, although precise timing of samples varied across participants (43). We surmise that this binary outcome does not capture potential drug effects and that quantitative culture of daily samples, particularly early treatment time points, is necessary to capture drug potency in vivo.

A further consideration of our analysis is selection of optimal virologic endpoints in clinical trials. The PLATCOV study demonstrates that viral clearance slope is an efficient and robust metric to identify potency after enrollment of a limited number of trial participants (4). It is advantageous relative to time to viral clearance, as it incorporates all viral load data points in the analysis. We observed small non–statistically significant differences between clearance slopes of total versus non-mutated viral RNA in simulations of all 3 trials. However, we observed significant reductions in viral AUC during therapy in all 3 simulated trials, suggesting that this may be an even more sensitive trial endpoint. In viral dynamic models, viral AUC maps directly to surface area of total infected cells, providing a mechanistic underpinning of why this may be a useful endpoint (44, 45).

Our results suggest that viral loads may vary according to drug level given molnupiravir’s short plasma half-life. A substudy within future trials comparing viral loads between drug trough and peak would be useful for the field. This could validate our model’s prediction that even small reductions in viral RNA may be associated with substantial reductions in total viral AUC particularly with a drug with a short half-life. Even minor reductions in viral load may be associated with substantial clinical benefit in this case. These fluctuations may be less evident if the intracellular half-life of the drug is longer or if PK measures in our model underestimate true drug levels in PANORAMIC due to older age or impaired renal clearance. Model accuracy would be improved if paired viral load and PK data were available from the same patients in similar trials.

Another key practical outcome is that, as for nirmatrelvir, extension of molnupiravir therapy to 10 days is likely to prevent rebound, although our simulations do not suggest any benefit from increasing dose (14). This suggests that prolonging therapy or using agents with a longer half-life is ideal for treating SARS-CoV-2 (46).

Each trial represented a unique set of issues for model fitting. In MOVe-OUT, because the mean viral kinetics curve of treatment arm differed only slightly from that of the control arm, the model without drug provided reasonable fit to the treatment arm. Nevertheless, the paf was identifiable for this trial, indicating that the model was able to detect and specify the limited potency of the drug. The fact that the drug’s potency and clinical efficacy appears to have increased with introduction of the Omicron variant demonstrates a massive challenge for the therapeutics field; as with vaccines (47), trials performed when prior variants were circulating may prove less relevant as new variants continually emerge. A priority should be retesting existing agents against newly emerging variants in small nimble trials such as PLATCOV, with viral load endpoints.

For PLATCOV, the model for the treatment arm matched the trial data precisely and identified the paf. The drug achieved nearly maximal observed viral load reduction in this trial. We identified a similar trend for PANORAMIC. It is notable that the model had the flexibility to account for different viral loads between these trials by predicting more rapid innate immune responses in PLATCOV which enrolled younger and healthier participants.

We arrived at similar estimated pafs in the PLATCOV and PANORAMIC trials, which agreed when using 2 separate methods: fitting to individual viral loads and fitting to mean viral load reduction trial endpoints. This suggests that our approach using well-matched in silico cohorts and fitting to population level outcomes produces reliable results (14). In many cases, it remains challenging for academic researchers to obtain individualized data from industry-sponsored trials. Therefore, it is important that the endpoint-fitting approach be considered when these are the only data publicly available.

Finally, our results highlight challenges in trial design associated with the selection of PCR assays and their corresponding LODs. Each trial reported results with a different LOD, which in turn impacts the degree of viral load decrease that can be observed. Initial viral loads were notably higher in PANORAMIC and MOVe-OUT than PLATCOV. The equivalent viral loads between PANORAMIC and PLATCOV may reflect a more sensitive PCR in the PANORAMIC study, as past immunity has consistently predicted lower viral loads and more rapid viral elimination for Omicron variants (15, 48). On the other hand, PANORAMIC viral loads could have been higher than in PLATCOV despite both enrolling Omicron infections due to an older and less healthy population or sampling from different anatomic compartments. Ideally, equivalent internationally standardized PCR quantitation and sample sites would be used across all trials.

Our study has a few limitations. The estimated paf is based on the in vitro assay data against the Delta variant in Calu-3 cells. In vitro EC_50_ is sensitive to assay conditions, including cell type, the VOC, the multiplicity of infection (MOI), and specific lab. In general, while necessary for the initial evaluation of the drug activity against a pathogen, the inability of in vitro assays to match in vivo conditions makes them an unreliable proxy for the potency of the drug in humans. This explains the necessity of incorporating the paf parameter when simulating an antiviral clinical trial using pharmacodynamic data. While the estimated value of paf is dependent on the PD data used to estimate the in vitro EC_50_, the calculated in vivo EC_50_ (= paf × [in vitro EC_50_]) is only a function of our model fit to clinical trial data. We chose to estimate the in vivo EC_50_ using the paf in order to quantify the extent to which the in vitro value may be misleading.

While similar paf values were estimated for PLATCOV and PANORAMIC trials, there are important differences in the design of these trials, including the sampling site, that can impact the estimated in vivo EC_50_. The in vivo efficacy of the drug could differ between different anatomic compartments. The only way to assess this hypothesis would be to separately fit the model to viral load from different anatomic sites collected from the same participants in the same trial. Lacking such data from each trial, we could only compare the paf across trials.

In our model, we assumed all mutated viruses are noninfectious. While most drug-induced random mutations reduce the fitness/viability of the virus, clustered infections with the molnupiravir mutation signature have been detected in regions where the drug was widely used, suggesting transmission (7). We justify this assumption in our definition of drug efficacy because improved fitness due to molnupiravir is a rare event that does not occur in most treated individuals. We base this conclusion on 3 trials, showing statistically significant viral load reduction. Nevertheless, while incomplete efficacy likely reflects subtherapeutic drug levels, it is also possible that some drug-induced mutations do not lower viral fitness. We do not see evidence that these mutations are associated with drug resistance in our modeling.

For individuals who do not clear the virus within the observed sampling period, our model occasionally predicts prolonged shedding. The late acquired immune response in our model is time dependent and responsible for late viral elimination. However, when we do not observe clearance within 15 days, the model’s projections are less reliable. While we lack confidence in any viral dynamic model’s ability to extrapolate beyond the observed data, this does not impact our estimation of the drug’s in vivo potency which is applied during the first 5 days of treatment when viral load data are abundant. Longer sampling periods are needed to accurately model the drivers of persistent infection, which is more common in severely immunocompromised individuals.

Another limitation is that PK parameters were estimated using the data from the plasma concentration of healthy individuals with the age range of 19–60 (mean 39.6) years old and we were not able to use an individualized PK model based on lack of drug level data in all 3 trials. The clearance rate of renally cleared drugs often increase with age (49). This implies that the paf may be larger in an older population, such as in PANORAMIC participants. Furthermore, we used the plasma concentration in the combined model to calculate the drug efficacy. However, using the drug’s intracellular concentration with a longer half-life, represented by the peripheral compartment of the PK model, could also likely lead to a larger estimated paf.

Finally, the lack of tissue drug-level data limits our PK model’s ability to provide a complete mechanistic link between intracellular drug levels and inhibition of viral replication. Our estimate of in vivo EC_50_ is intended to provide a practical target for drug developers, as plasma drug levels are routinely and easily measured. However, it is important to note that the in vivo EC_50_ estimate within infected cells, which is the most relevant mechanistic value, cannot be estimated from existing data and may differ from our estimate of the plasma in vivo EC_50_.

In summary, we further demonstrate the utility of clinical trial simulation using models that capture drug PK and PD, as well as infection dynamics. In the case of molnupiravir, our results suggest that final viral endpoints should be adjusted based on the drug’s mechanism of action.

## Methods

### Sex as biological variable.

We used data from 3 trials in which both biological sexes were represented equally. In treatment arms, percentage of female and male participants were 53.6% and 46.4% in MOVe-OUT; 57% and 43% in PLATCOV; and 55% and 45% in PANORAMIC. In control arms, percentage of female and male participants were 49% and 51% in MOVe-OUT; 68% and 32% in PLATCOV; and 65% and 35% in PANORAMIC. We did not use biological sex as a covariate in our model.

### Study design.

We developed viral dynamic models recapitulating viral loads from symptomatic individuals in the NBA cohort (15). We used a 2-compartment model to reproduce PK data of molnupiravir (22). For clinical trial simulation, we constructed a virtual cohort by randomly selecting 400 individuals from the NBA cohort, matching trial populations regarding vaccine status and history of infection given cohort characteristics. Due to lack of individual PK data, we used estimated population PK parameters for all individuals in the virtual cohort. PD parameters for each individual were randomly selected from a log-normal distribution with means and standard errors estimated based on in vitro assay data. We first fit the combined viral dynamic and PKPD model to average change in viral load from baseline of control and treatment arms of 3 previously published molnupiravir clinical trials (2–4, 50). Comparing output to control arms validated our viral dynamic model and demonstrated how well our virtual cohorts represent trial control arms. We used average data from the treatment arms to estimate the paf by maximizing *R*^2^ of the fit (14). We next fit to individual viral load trajectories in PLATCOV and PANORAMIC using the mixed-effect population approach in Monolix (51–53) and obtained individual paf values and a population distribution.

### Viral load data.

The NBA cohort dataset published by Hay et al. consists of 2875 SARS-CoV-2 infections in 2678 people detected through frequent PCR testing regardless of symptoms (20). We used viral load data from 1510 infections in 1440 individuals with at least 4 positive quantitative samples to estimate viral dynamic parameters. We used parameter sets estimated for the symptomatic subpopulation of these individuals to construct virtual cohorts (15).

### Clinical trial data.

We used viral load data from three molnupiravir trials. MOVe-OUT included 544 and 549 symptomatic high-risk individuals in control and treatment arms, respectively (2). We obtained average change in viral load data of the control and treatment arms as shared in Table 1 or in the supplemental material of Jayk Bernal et al. (2). Nasal viral load was measured using PCR assay on days 0, 3, 5, 10, and 14 after treatment start day and adjusted by baseline viral load. An LOD of 500 copies/mL was used in this trial. Treatment was started within 5 days of symptom onset.

PLATCOV was an open-label, randomized, controlled adaptive trial with 85 and 58 symptomatic, young, healthy individuals in control and molnupiravir treatment arms (4). Oropharyngeal samples from each participant were collected daily on days 0 through 7 and on day 14 after treatment start. Viral load was measured using PCR. We used individual viral load data for model fitting. From PLATCOV, we averaged 2 oral samples collected from each individual for individual-level viral load fitting and then calculated viral load drop from baseline for the trial endpoint-fitting approach. When comparing simulation results with data we used the maximum LOD reported in the trial (~1.26 log).

PANORAMIC was a platform adaptive randomized, controlled trial with 42 and 38 symptomatic, vaccinated individuals with at least one risk factor in the control and treatment arms, respectively (3). Nasal viral load was measured using PCR. Samples were collected on days 0 through 6 and on day 13. We used individual viral load data shared by the authors and adjusted by baseline viral load to obtain mean drop from baseline. Mean days since symptom onset at baseline were 2.4 (SD 0.78) for treatment arm and 2.5 (SD 1.12) for control arm. The LOD of 100 copies/mL (imputed as 50 copies/mL) was used.

In all 3 trials, study participants were treated with 800 mg of molnupiravir twice per day, for 5 days.

### PK data.

Mean plasma concentration data of molnupiravir were obtained by digitizing Figure 3 of a phase I trial using WebPlotDigitizer (22). In the study, 6 participants were given 50, 100, 200, 300, 400, 600, and 800 mg of molnupiravir twice daily for 5.5 days. Plasma concentrations were measured after the first and last doses.

### PD data.

Data on drug efficacy were obtained from experiments performed at the University of Washington. Efficacy of molnupiravir was measured against the Delta variant of SARS-CoV2 in Calu-3 cells (human lung epithelial). Cells were treated with varying concentrations of molnupiravir prior to infection with SARS-CoV-2 at an MOI of 0.01. Antiviral efficacy and cell viability (of non-infected cells treated with drugs) were assessed after 96 hours of incubation using the CellTiter-Glo assay, which measures number of viable cells in culture by quantifying ATP (9, 14). There were 5 replicates per condition, pooled from 2 independent technical repeats (one experiment with triplicate conditions, one with duplicate conditions).

### Viral dynamics model.

We used a data-validated model of SARS-CoV-2 dynamics to model viral load of symptomatic individuals with SARS-CoV-2 infection (15). This model assumes susceptible cells are infected at rate *β*SV by virions. Infected cells go through a non-productive eclipse phase (I_E_) before transitioning at rate *κ*I_E_ to productively infected cells (I_p_). When encountering productively infected cells, susceptible cells become refractory to infection (R) at the rate *Φ*I_p_S. Refractory cells revert to susceptible rate *ρ*R. Productively infected cells produce virus at rate *π*I_p_ and are cleared at rate *δ*I, representing cytolysis and innate immune responses that lack memory and are proportional to the amount of ongoing infection. If infection persists longer than time *τ*, then cytotoxic acquired immunity is activated, which is represented in our model by rate *m*I_p_. Finally, free virions are cleared at rate *γ*. This model, previously proposed by Ke et al. was selected against other models based on superior fit to data and parsimony (54). The set of differential equations has the following form:

*d*S/*dt* = –*β*SV – *Φ*I_p_S + *ρ*R (Equation 1A)

*d*R/*dt* = *Φ*I_p_S – *ρ*R (Equation 1B)

*d*I_E_/*dt* = *β*SV – *κ*I_E_ (Equation 1C)

*d*I_p_/*dt* = *κ*I_E_ – *Φ*I_p_ – *m*(*t*)I_p_ (Equation 1D)

*d*V/*dt* = *π*I_p_ – *γ*V (Equation 1E)

 (Equation 1F)
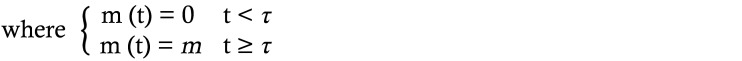


To estimate parameter values for the VID model prior to application of treatment, we fit it to viral loads from the NBA cohort using a mixed-effect population approach implemented in Monolix. Details on model selection and fitting can be found in Owens et al. (15).

For subsequent concurrent model fit to data from the NBA cohorts and trial participants, we started all simulations at the beginning of infection (*t* = –*t*_0_) with susceptible cells. The parameter was estimated as time of infection relative to earliest record of infection in the data (*t* = 0). In the NBA cohort, because time of symptom onset was not known for all individuals, *t* = 0 was set as time of first recorded positive test. In the clinical trials, *t* = 0 was set as the time of symptom onset, as time of sample collection was measured relative to time of symptom onset. Mean was approximately 12–14 hours higher in the NBA cohort (2.16 days) relative to the trials (1.6 days for PLATCOV and 1.54 days for PANORAMIC) because in the NBA cohort sampling did not always occur daily in the absence of infection. We suspect values for the 2 trials were slightly shorter than observed incubation periods for Omicron (2–3 days) because they do not represent precise time of exposure, but rather the time at which viral infection of cells initiates. While mean duration of infection at first obtained sample was 2.16 days in the NBA cohort, it was 3.63 days for PLATCOV and 3.59 days for PANORAMIC, reflecting a 2.0-day average delay in sampling and starting treatment attributable to the trial enrollment process. This 1.5-day difference highlights why NBA participant data was useful to inform modeling of early presymptomatic, expansion time points.

At *t* = –*t*_0_, the value of refractory cells was assumed to be zero since interferon signaling was not likely to be active. We assumed no infected cells (eclipse or productive) at infection initiation. We fixed the level of inoculum (V_0_) at for each individual due to little individual variability when this parameter was previously estimated. The selected value (97 copies/mL) was our previous population estimate (15). Simulations starting with a small number of cells in eclipse or productively infected phase allowed similar viral kinetics.

To resolve identifiability issues and based on information from our prior extensive fitting, we fixed 2 parameter values, setting the inverse of the eclipse phase duration to days, and the rate of viral clearance to *γ* = day^–1^ (15).

### PK model.

We used a 2-compartment PK model that includes amount of drug in the GI tract (A_GI_), plasma compartment (A_p_), and respiratory tract (A_L_). The drug is administered orally, passes through the GI tract, and gets absorbed into blood at rate *κ_a_*. The drug transfers from the blood into the peripheral compartment (or respiratory tract) at rate. The metabolized drug transfers back into plasma at rate from where it clears from the body at rate. The ordinary differential equations are:

*d*A_GI_/*dt* = –*κ_a_*A_GI_ (Equation 2A)

*d*A_p_/*dt* = *κ_a_*A_GI_ + *κ_LP_*A_L_ – (*κ_CL_* + *κ_PL_*)A_p_ (Equation 2B)

*d*A_L_/*dt* = *κ_PL_*A_p_ – *κ_LP_*A_L_ (Equation 2C)

We used Monolix and a mixed-effect population approach to estimate parameters and their standard deviations. With initial condition of (A_GI_ = Dose, A_p_ = 0, A_L_ = 0), we fit C_p_ = A_p_/Vol to plasma concentration data, where Vol is estimated plasma volume. Details on parameter values and the error model are in Supplemental Table 1.

### PD model.

We used a Hill equation ε(*t*) = *E_max_*C(*t*)*^n^*/(C(*t*)*^n^* + EC_50_*^n^*), where C(*t*) is drug concentration in plasma, *E_max_* is maximum efficacy, *n* is the Hill coefficient, and EC_50_ is drug concentration in plasma required for 50% efficacy. We used least-squared fitting to obtain 3 parameters (*E_max_*, *n*, and EC_50_) and their SDs. Average drug efficacy over treatment period was calculated using Equation 3:

 (Equation 3)
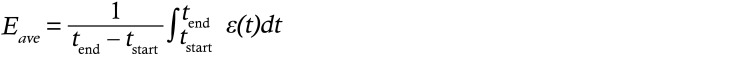


where *t*_start_ and *t*_end_ are treatment start and end day, respectively.

### Combined PKPD and VL models.

The plasma concentration of molnupiravir obtained from the PK model is used in the PD model to obtain time-dependent efficacy. Since molnupiravir imposes lethal mutations during viral replication, in our model, a portion of all viruses produced by an infected cell, measured by *ε*(*t*), are mutated (V_m_) and assumed to be non-infectious, with the addition that most detected viral RNA pretreatment is also non-infectious. The production rate of non-mutated viruses is decreased by a factor of 1 – *ε*(*t*). Equation 1E is thus replaced with:

*d*V/*dt* = (1 – *ε*(*t*)*π*I_p_ – *γ*V (Equation 4A)

*d*V_m_/*dt* = *ε*(*t*)*π*I_p_ – *γ*V_m_ (Equation 4B)

Total viral load (V + V_m_) was used to fit the PCR assay data from each trial.

### Potency adjustment factor (paf).

The paf (prf in our previous work) (14) is defined as:

paf = EC_50_ in vivo/EC_50_ in vitro (Equation 5)

We changed prf (potency reduction factor) to paf (potency adjustment factor), since our model showed that in vivo potency can be higher than in vitro, which we did not initially expect.

### Fitting the combined model to individual viral load data in the PLATCOV and PANORAMIC trials.

We used the population mixed-effect approach implemented in Monolix to estimate each individual’s viral dynamics parameters and paf. The individual viral load data at time *k* is modeled as:

log_10_(V_ik_) = *M*(*t_k_*, *θ_i_*) + *aε* (Equation 6)

where *M*(*t_k_*, *θ_i_*) is the solution of the combined VL+PKPD ODE model, *θ_i_* = [*β_i_*, *θ_i_*, *ρ_i_*, *κ_i_*, *δ_i_*, *m_i_*, *π_i_*, *γ_i_*, *τ_i_*, *κ_a_*, *κ_LP_*, *κ_CL_*, *η*, *EC_50_*, *paf_i_*] is the parameter vector for individual *i*, *ε*~*N*(0,1) is measurement error of viral load data, and *a* is the magnitude of the measurement error known as the error parameter. The parameters of individual *i* is modeled as *θ_i_* = *θ_pop_* + *η_i_*, if *θ_i_* can take positive and negative values and is normally distributed, or as *θ_i_* = *θ_pop_*exp (*η_i_*) if the parameter can only take positive values and is log-normally distributed. *θ_pop_* is average population value of the parameters and *η_i_* known as the random effect is an individual *i*’s deviation from average.

Due to lack of data from the initial phase of infection in PLATCOV and PANORAMIC, we included data from Omicron-infected individuals in the NBA cohort in the fitting population to inform the model about the initial phase of infection. We fixed PK parameters to the estimated population values (Supplemental Table 1) and PD parameters, including the in vitro EC_50_, to in vitro estimated population values (Supplemental Table 2). We used the study category (NBA vs. PLATCOV and PANORAMIC) as a covariate for *t*_0_ (estimated timing of infection) for reasons described above and τ (timing of the adaptive immune response). This accounted for discrepancies between the definition of *t* = 0 between the NBA cohort and trials.

### Construction of a virtual cohort.

To generate a cohort for our simulated clinical trials, we randomly selected 400 individuals (for each arm) from the unvaccinated symptomatic subpopulation of the NBA cohort for MOVe-OUT and the vaccinated, Omicron-infected subpopulation for PLATCOV and PANORAMIC and used their individual viral load parameters estimated by fitting our viral dynamics model to the data, using population mixed-effect model, as described above. A sample size of *n* = 400 was used to mimic a large-scale clinical trial and maintain relatively low overlap between virtual cohorts used in each arm of the simulations and between different simulations. Since time of symptom onset is not available for all individuals in the NBA data, we randomly drew an incubation period for each individual from gamma distributions with variant-specific parameters estimated by Gamiche et al. (55). The start of treatment relative to symptom onset was randomly selected according to a uniform distribution for MOVe-OUT and PLATCOV, and a log-normal distribution for PANORAMIC with limits of [0,5] days and mean and SD reported in the PANORAMIC trial for control and treatment arms. The same population PK parameters were assigned to each individual. The relevant dose in each scenario was added to the A_GI_ compartment (the absorption equation) of the PK model (Equation 2A) at each dosing time point (*t* = 0, 0.5, 1, 1.5, …., 4.5 days). For each dose, appropriate PK parameters were used (Supplemental Table 1). PD parameters were randomly drawn from a log-normal distribution with estimated mean and SD. The SD of the PD parameters represents the accuracy of the assays and not individual variability.

We estimated the paf by maximizing *R^2^*, the measure of agreement between the drop in viral load of the treatment arm of our simulation and the change in viral load observed in the treatment arm of the clinical trial on day 0 through day 7.

### Measuring rebound probability.

Viral load rebound in the treatment arm was defined when viral load at any time after treatment ended exceeded viral load at the end of treatment by 1 on a log_10_ scale. In the control group, viral rebound was defined as at least 2 peaks with minimum height of 1000 copies/mL with the second peak 1 log_10_ higher than its preceding local minimum.

### Statistics.

We used the 2-sided Kolmogorov-Smirnov test to compare observed and simulated viral load drop distributions at each time point. Adjusted *P* values were calculated using the Benjamini-Hochberg method and represent dissimilarity between distributions. When comparing treated and untreated total and non-mutated viral load at a given time point, viral AUC, viral clearance slope, and viral dynamics parameters between different trial arms, we used the 2-sided Mann-Whitney *U* test with Bonferroni’s correction. *P* values of less than 0.05 were considered statistically significant.

### Study approval.

No new human or animal data were collected. All data analyzed were deidentified prior to our use and previously published.

### Data availability.

Values for all data points in graphs are reported in the Supporting Data Values file. The data analyzed in this work was previously published by Hay et al., Schilling et al., and Standing et al. and are available at https://github.com/gradlab/SC2-kinetics-immune-history, https://github.com/jwatowatson/PLATCOV-Molnupiravir/tree/V1.0, and https://zenodo.org/records/10375295 PD data are available on GitHub at https://github.com/sEsmaeili/MolnupiravirModeling All codes and materials used in the analysis are available on GitHub (https://github.com/sEsmaeili/MolnupiravirModeling).

## Author contributions

JTS, SE, and KO conceptualized the study. JTS, SE, and KO developed the modeling methods. SE and KO implemented the modeling software. JTS, SE, SJP, and JW performed experimental and modeling investigation. SE formally analyzed model data. JTS and SE wrote the original manuscript draft. JTS, SE, KO, SJP, JFS, DML, SZ, JAW, WHKS, and UAPL reviewed and edited the manuscript.

## Funding support

This work is the result of NIH funding, in whole or in part, and is subject to the NIH Public Access Policy. Through acceptance of this federal funding, the NIH has been given a right to make the work publicly available in PubMed Central.

- National Institute of Allergies and Infectious Diseases grant R01AI77512-01 (to JTS and SP).

## Supplementary Material

Supplemental data

Supporting data values

## Figures and Tables

**Table 1:**
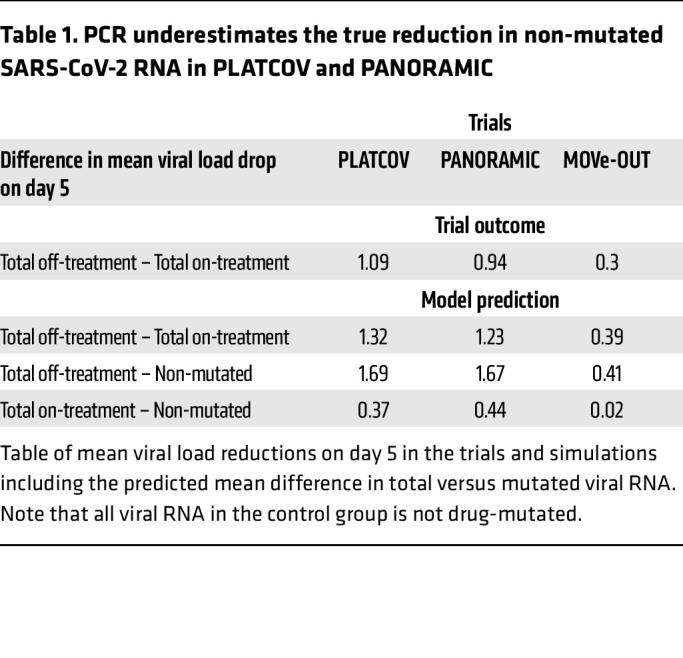
PCR underestimates the true reduction in non-mutated SARS-CoV-2 RNA in PLATCOV and PANORAMIC

## Version 1 09/11/2025

In-Press Preview

## Version 2 11/03/2025

Electronic publication

## References

[1] Abdelnabi R (2021). Molnupiravir inhibits replication of the emerging SARS-CoV-2 variants of concern in a hamster infection model. J Infect Dis.

[2] Jayk Bernal A (2022). Molnupiravir for oral treatment of Covid-19 in nonhospitalized patients. N Engl J Med.

[3] Butler CC (2023). Molnupiravir plus usual care versus usual care alone as early treatment for adults with COVID-19 at increased risk of adverse outcomes (PANORAMIC): an open-label, platform-adaptive randomised controlled trial. Lancet.

[4] Schilling WHK (2024). Antiviral efficacy of molnupiravir versus ritonavir-boosted nirmatrelvir in patients with early symptomatic COVID-19 (PLATCOV): an open-label, phase 2, randomised, controlled, adaptive trial. Lancet Infect Dis.

[5] Hammond J (2022). Oral nirmatrelvir for high-risk, nonhospitalized adults with Covid-19. N Engl J Med.

[6] Fountain-Jones NM (2024). Effect of molnupiravir on SARS-CoV-2 evolution in immunocompromised patients: a retrospective observational study. Lancet Microbe.

[7] Sanderson T (2023). A molnupiravir-associated mutational signature in global SARS-CoV-2 genomes. Nature.

[8] Rosenke K (2023). Combined molnupiravir-nirmatrelvir treatment improves the inhibitory effect on SARS-CoV-2 in macaques. JCI Insight.

[9] Wagoner J (2022). Combinations of host- and virus-targeting antiviral drugs confer synergistic suppression of SARS-CoV-2. Microbiol Spectr.

[10] Cox RM (2023). Comparing molnupiravir and nirmatrelvir/ritonavir efficacy and the effects on SARS-CoV-2 transmission in animal models. Nat Commun.

[11] Iranzo J (2011). Tempo and mode of inhibitor-mutagen antiviral therapies: a multidisciplinary approach. Proc Natl Acad Sci U S A.

[12] Łagocka R (2021). Favipiravir in therapy of viral infections. J Clin Med.

[13] Neogi U (2020). Feasibility of known RNA polymerase inhibitors as anti-SARS-CoV-2 drugs. Pathogens.

[14] Esmaeili S (2024). A unifying model to explain frequent SARS-CoV-2 rebound after nirmatrelvir treatment and limited prophylactic efficacy. Nat Commun.

[15] Owens K (2024). Heterogeneous SARS-CoV-2 kinetics due to variable timing and intensity of immune responses. JCI Insight.

[16] Edelstein GE (2023). SARS-CoV-2 virologic rebound with nirmatrelvir-ritonavir therapy: an observational study. Ann Intern Med.

[17] Harrington PR (2023). Evaluation of SARS-CoV-2 RNA rebound after nirmatrelvir/ritonavir treatment in randomized, double-blind, placebo-controlled trials - United States and International sites, 2021-2022. MMWR Morb Mortal Wkly Rep.

[18] Anderson AS (2022). Nirmatrelvir-ritonavir and viral load rebound in Covid-19. N Engl J Med.

[20] Hay JA (2022). Quantifying the impact of immune history and variant on SARS-CoV-2 viral kinetics and infection rebound: a retrospective cohort study. Elife.

[21] https://arxiv.org/abs/2102.11543.

[22] Painter WP (2021). Human safety, tolerability, and pharmacokinetics of molnupiravir, a novel broad-spectrum oral antiviral agent with activity against SARS-CoV-2. Antimicrob Agents Chemother.

[23] Shen L (2008). Dose-response curve slope sets class-specific limits on inhibitory potential of anti-HIV drugs. Nat Med.

[24] Lieber CM (2022). SARS-CoV-2 VOC type and biological sex affect molnupiravir efficacy in severe COVID-19 dwarf hamster model. Nat Commun.

[25] Vangeel L (2022). Remdesivir, molnupiravir and nirmatrelvir remain active against SARS-CoV-2 Omicron and other variants of concern. Antiviral Res.

[26] Akın E (2020). Continuous and discrete modeling of HIV-1 decline on therapy. J Math Biol.

[27] Dixit NM (2004). Modelling how ribavirin improves interferon response rates in hepatitis C virus infection. Nature.

[28] Zhou S (2024). Combined treatment of severe acute respiratory syndrome coronavirus 2 reduces molnupiravir-induced mutagenicity and prevents selection for nirmatrelvir/ritonavir resistance mutations. J Infect Dis.

[29] Schiffer JT (2013). Rapid viral expansion and short drug half-life explain the incomplete effectiveness of current herpes simplex virus 2-directed antiviral agents. Antimicrob Agents Chemother.

[30] Schiffer JT (2016). Mathematical modeling of herpes simplex virus-2 suppression with pritelivir predicts trial outcomes. Sci Transl Med.

[31] Backer JA (2022). Shorter serial intervals in SARS-CoV-2 cases with Omicron BA.1 variant compared with Delta variant, the Netherlands, 13 to 26 December 2021. Euro Surveill.

[32] Wu Y (2022). Incubation period of COVID-19 Caused by unique SARS-CoV-2 strains: a systematic review and meta-analysis. JAMA Netw Open.

[33] Li P (2022). SARS-CoV-2 Omicron variant is highly sensitive to molnupiravir, nirmatrelvir, and the combination. Cell Res.

[34] Yotsuyanagi H (2024). Efficacy and safety of 5-day oral ensitrelvir for patients with mild to moderate COVID-19: the SCORPIO-SR randomized clinical trial. JAMA Netw Open.

[35] Cruciani M (2024). SARS-CoV-2 infection rebound among patients receiving antiviral agents, convalescent plasma, or no treatment: a systematic review with meta-analysis. Blood Transfus.

[36] Reeves DB (2023). High monoclonal neutralization titers reduced breakthrough HIV-1 viral loads in the antibody mediated prevention trials. Nat Commun.

[37] Nguyen BT (2025). A viroimmunologic model to characterize the antiviral effect of molnupiravir in outpatients infected with SARS-CoV-2: implication for treatment duration. J Infect Dis.

[38] Kabinger F (2021). Mechanism of molnupiravir-induced SARS-CoV-2 mutagenesis. Nat Struct Mol Biol.

[39] Menéndez-Arias L (2021). Decoding molnupiravir-induced mutagenesis in SARS-CoV-2. J Biol Chem.

[40] Gordon CJ (2021). Molnupiravir promotes SARS-CoV-2 mutagenesis via the RNA template. J Biol Chem.

[41] Alteri C (2022). A proof-of-concept study on the genomic evolution of Sars-Cov-2 in molnupiravir-treated, paxlovid-treated and drug-naïve patients. Commun Biol.

[42] Reeves DB (2023). Impact of misclassified defective proviruses on HIV reservoir measurements. Nat Commun.

[43] Standing JF (2024). Randomized controlled trial of molnupiravir SARS-CoV-2 viral and antibody response in at-risk adult outpatients. Nat Commun.

[44] Schiffer JT, Corey L (2013). Rapid host immune response and viral dynamics in herpes simplex virus-2 infection. Nat Med.

[45] Schiffer JT (2010). Mucosal host immune response predicts the severity and duration of herpes simplex virus-2 genital tract shedding episodes. Proc Natl Acad Sci U S A.

[46] Weinreich DM (2021). REGN-COV2, a neutralizing antibody cocktail, in outpatients with Covid-19. N Engl J Med.

[47] Feikin DR (2022). Duration of effectiveness of vaccines against SARS-CoV-2 infection and COVID-19 disease: results of a systematic review and meta-regression. Lancet.

[48] Watson JA (2022). Characterizing SARS-CoV-2 viral clearance kinetics to improve the design of antiviral pharmacometric studies. Antimicrob Agents Chemother.

[49] Lonsdale DO (2020). β-Lactam antimicrobial pharmacokinetics and target attainment in critically ill patients aged 1 day to 90 years: the ABDose study. J Antimicrob Chemother.

[50] Singh S (2024). The relationship between viral clearance rates and disease progression in early symptomatic COVID-19: a systematic review and meta-regression analysis. J Antimicrob Chemother.

[51] Prague M, Lavielle M (2022). SAMBA: a novel method for fast automatic model building in nonlinear mixed-effects models. CPT Pharmacometrics Syst Pharmacol.

[52] Bing A (2020). Comparison of empirical and dynamic models for HIV viral load rebound after treatment interruption. Stat Commun Infect Dis.

[53] Chan PL (2011). The use of the SAEM algorithm in MONOLIX software for estimation of population pharmacokinetic-pharmacodynamic-viral dynamics parameters of maraviroc in asymptomatic HIV subjects. J Pharmacokinet Pharmacodyn.

[54] Ke R (2021). In vivo kinetics of SARS-CoV-2 infection and its relationship with a person’s infectiousness. Proc Natl Acad Sci U S A.

[55] Galmiche S (2023). SARS-CoV-2 incubation period across variants of concern, individual factors, and circumstances of infection in France: a case series analysis from the ComCor study. Lancet Microbe.

