# Supplementary material for: Molnupiravir clinical trial simulation suggests that polymerase chain reaction underestimates antiviral potency against SARS-CoV-2
Source: J Clin Invest. 2025 Aug 28;135(21):e192052. doi: 10.1172/JCI192052 (PMC12578382; doi:10.1172/JCI192052)
Supplement: Supplemental data [file jci-135-192052-s358.pdf]

Supplementary Materials

For

Molnupiravir clinical trial simulation suggests that polymerase chain reaction underestimates antiviral potency against SARS-CoV-2

Authors

Shadisadat Esmaeili[1]*, Katherine Owens[1], Ugo Avila-Ponce de Leon[1] , Joseph F. Standing[2,3], David M. Lowe[4,5], Shengyuan Zhang[2], James A. Watson[6,7], William H. K. Schilling[7,8], Jessica Wagoner[9], Stephen J. Polyak[9], Joshua T. Schiffer[1,10]

Affiliations

[1] Vaccine and Infectious Disease Division, Fred Hutchinson Cancer Center; Seattle, WA, USA.

[2] Infection, Immunity and Inflammation, Great Ormond Street Institute of Child Health, University College London, London, UK

[3] Great Ormond Street Hospital for Children NHS Trust, London, UK

[4] Department of Clinical Immunology, Royal Free London NHS Foundation Trust, London, UK

[5] Institute of Immunity and Transplantation, University College London, London, UK

[6] Infectious Diseases Data Observatory, Oxford, UK

[7] Centre for Tropical Medicine and Global Health, Nuffield, Department of Medicine, University of Oxford, Oxford, UK

[8] Mahidol Oxford Tropical Medicine Research Unit, Bangkok, Thailand

[9] Department of Laboratory Medicine & Pathology, University of Washington; Seattle, WA, USA

[10] Department of Medicine, University of Washington; Seattle, WA, USA.

*Corresponding Author: sesmaeil@fredhutch.org

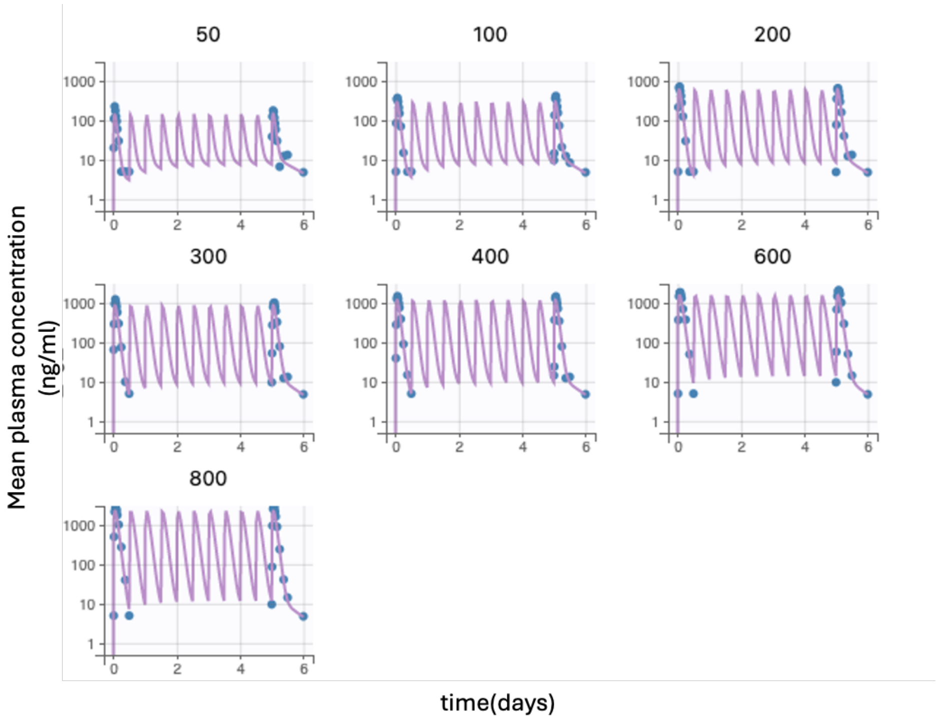

Figure S1. Pharmacokinetics model fit to data. Datapoints reflect mean plasma concentration of 50, 100, 200, 300, 400, 600, and 800 mg of molnupiravir given twice daily for 5.5 days. Model output is puple line.

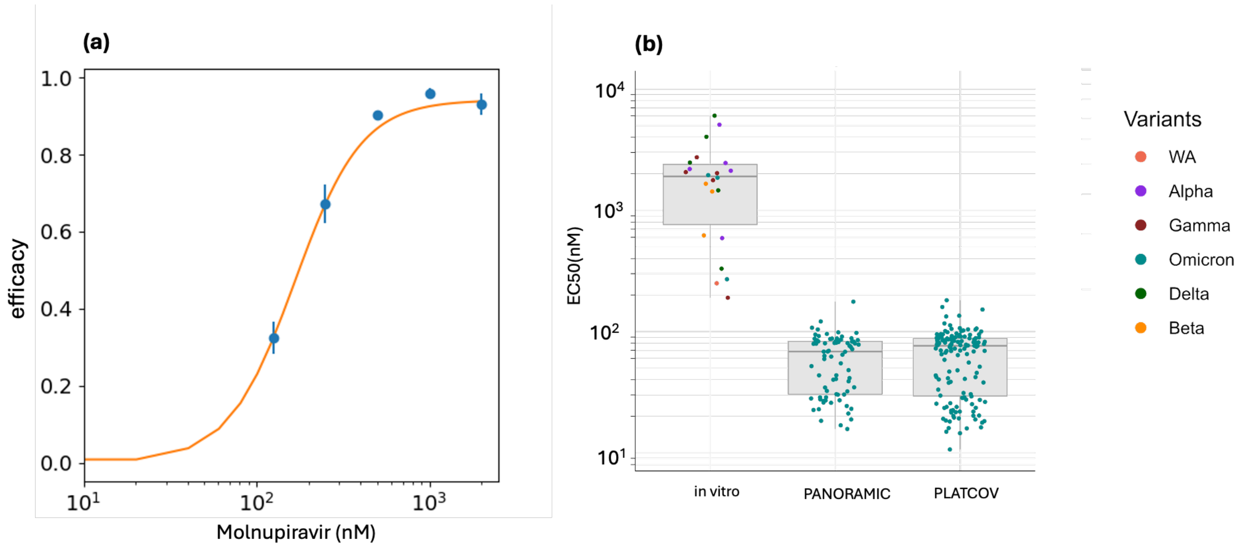

Figure S2. Pharmacodynamic model fit to in-vitro assay data. a) The estimated EC50 is 177 nMOL based on model fit (orange line) to our internal dose response data (blue dots). b) Review of *in vitro* EC50 estimates from the literature labelled according to variant of concern.

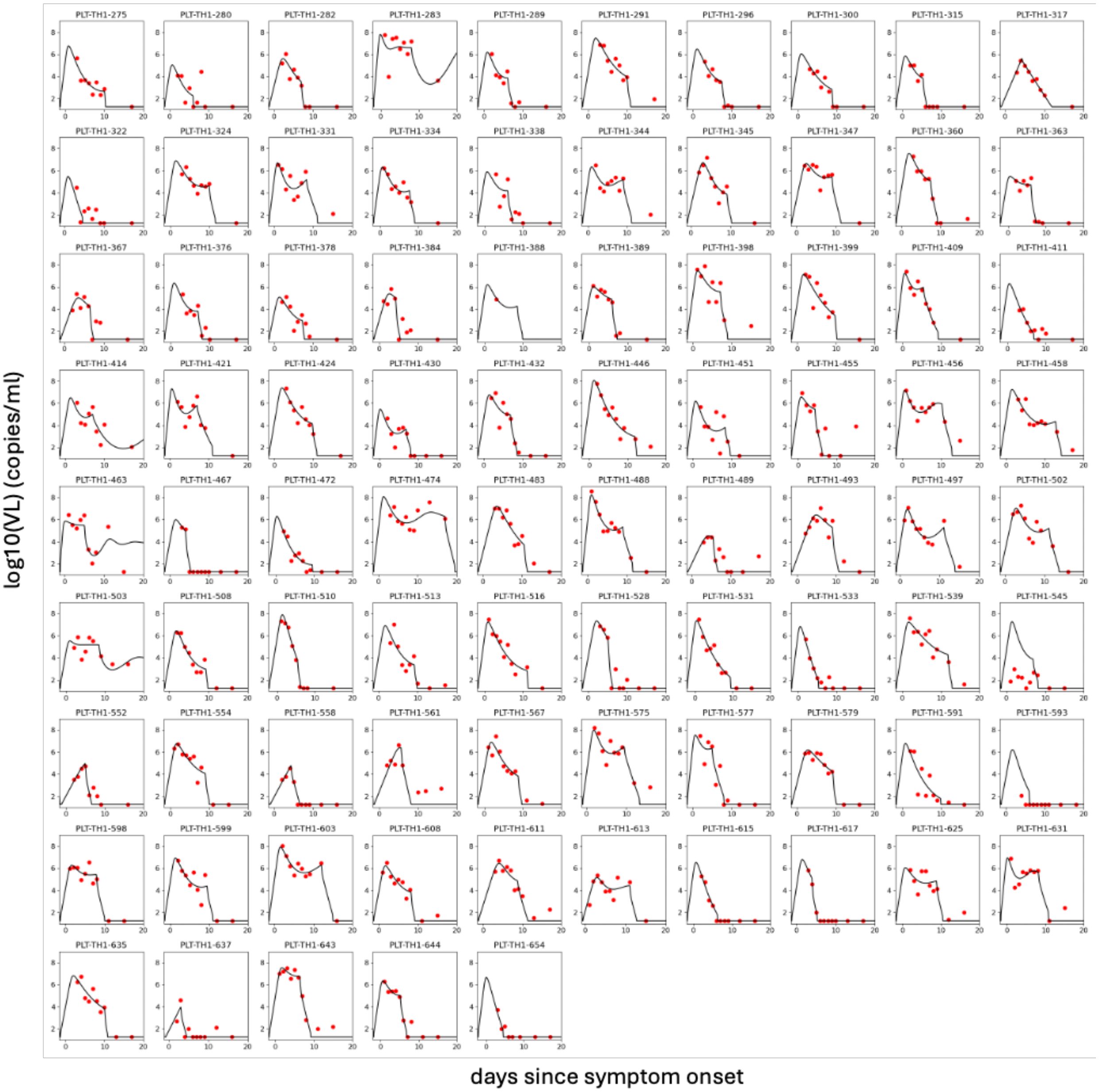

Figure S3. Individual model fits to viral load data from the control arm of PLATCOV trial. Dots are participant viral loads while the black line is model outout.

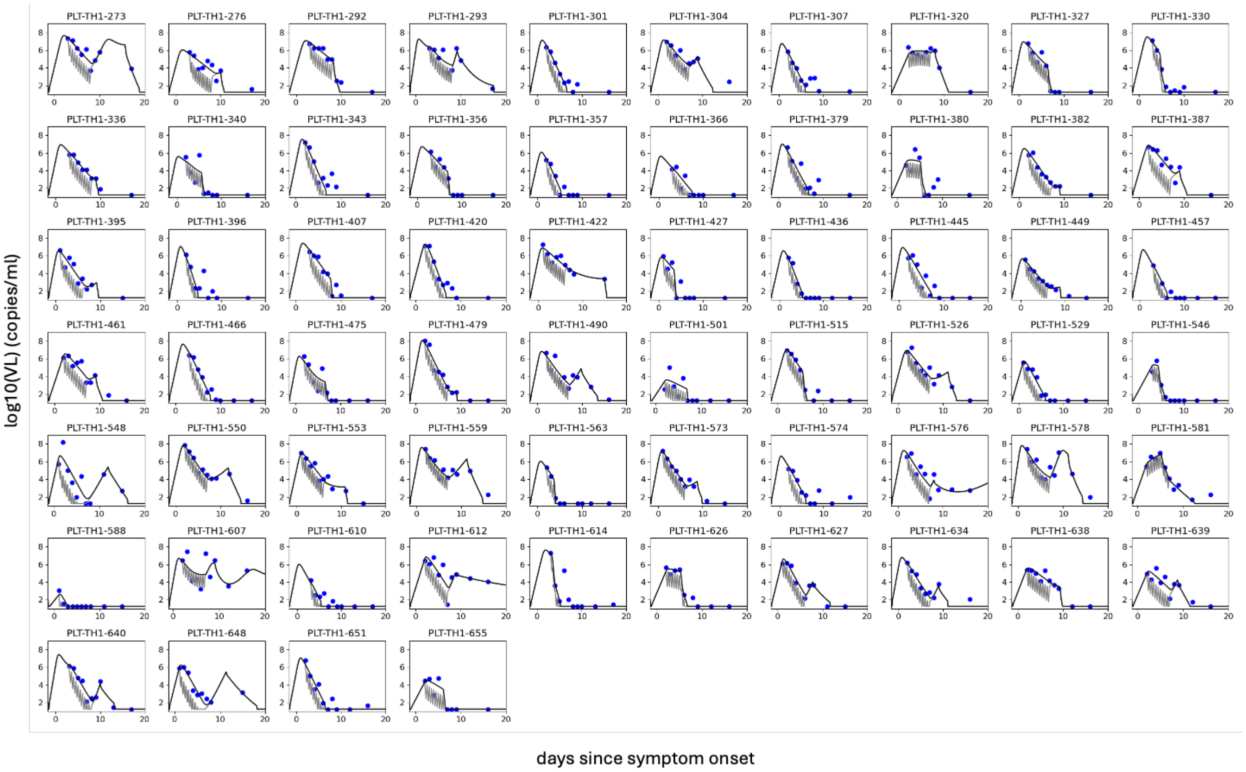

Figure S4. Individual model fits to viral load data from the molnupiravir treatment arm of the PLATCOV trial. Dots are participant viral loads. The top black line is model outout for viral RNA while the lower black line is simulated non-drug mutated viral RNA.

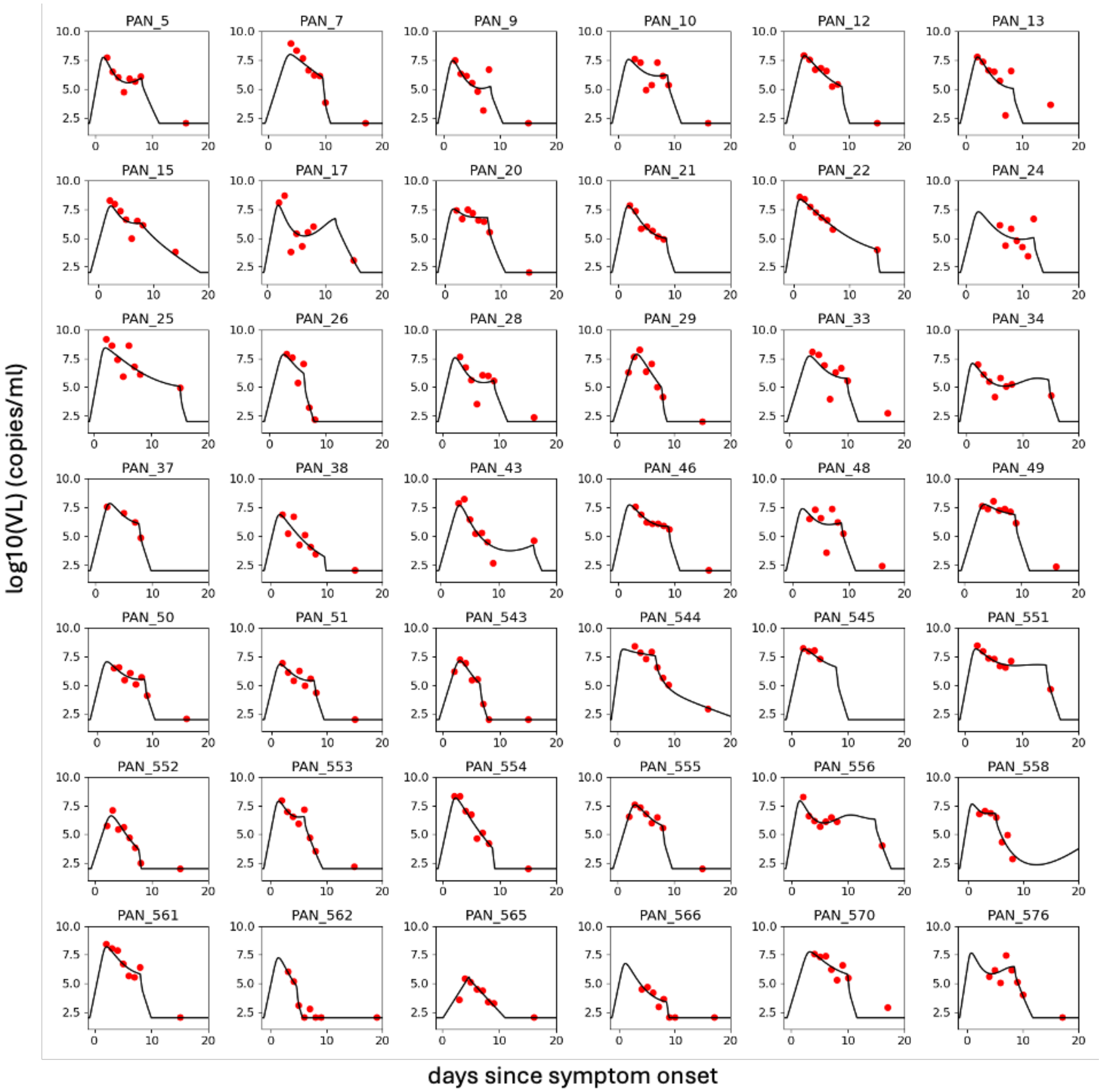

Figure S5. Individual model fits to viral load data from the control arm of PANORAMIC trial. Dots are participant viral loads while the black line is model outout.

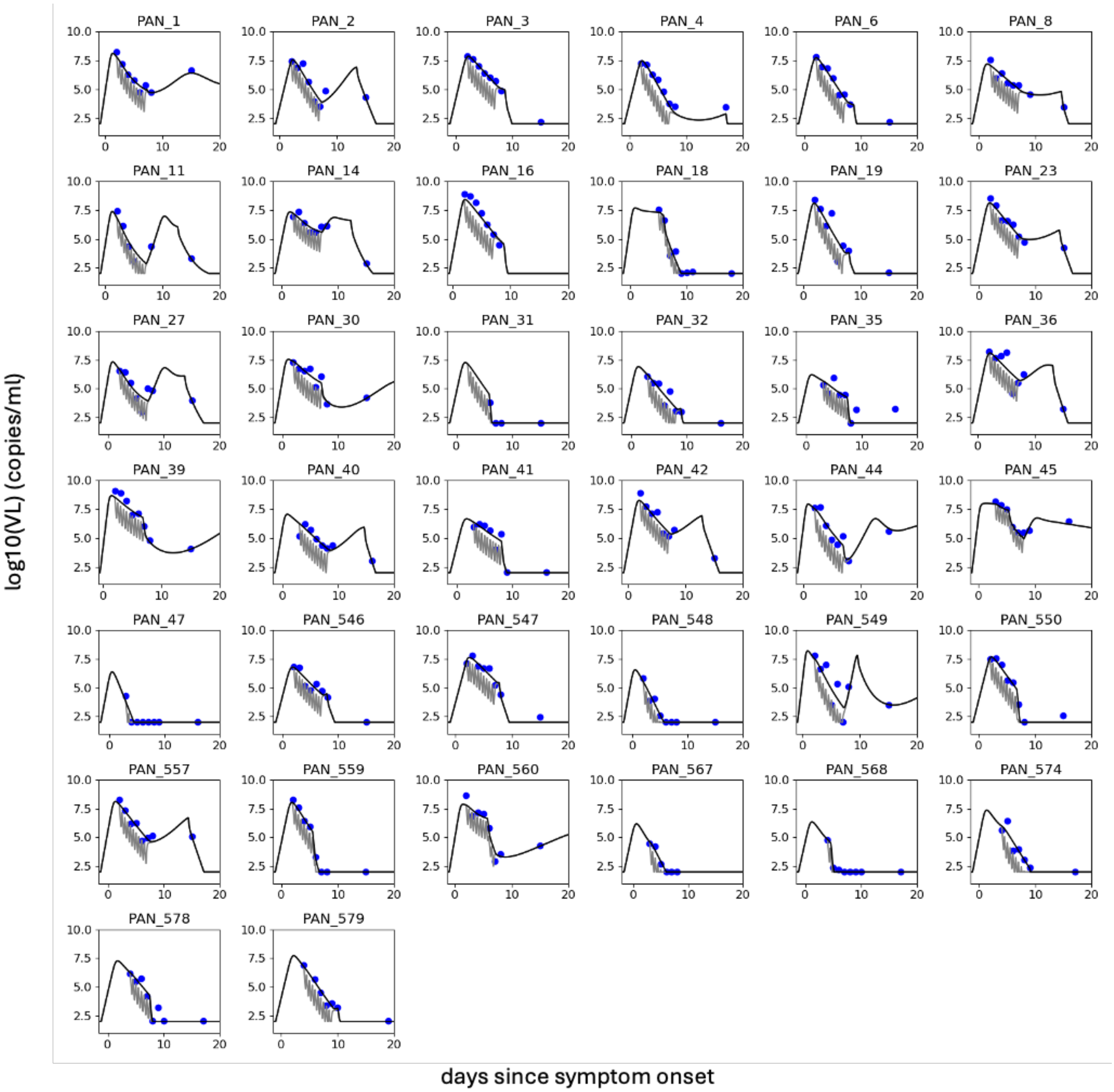

Figure S6. Individual model fits to viral load data from the treatment arm of PANORAMIC trial. Dots are participant viral loads. Dots are participant viral loads. The top black line is model outout for viral RNA while the lower black line is simulated non-drug mutated viral RNA.

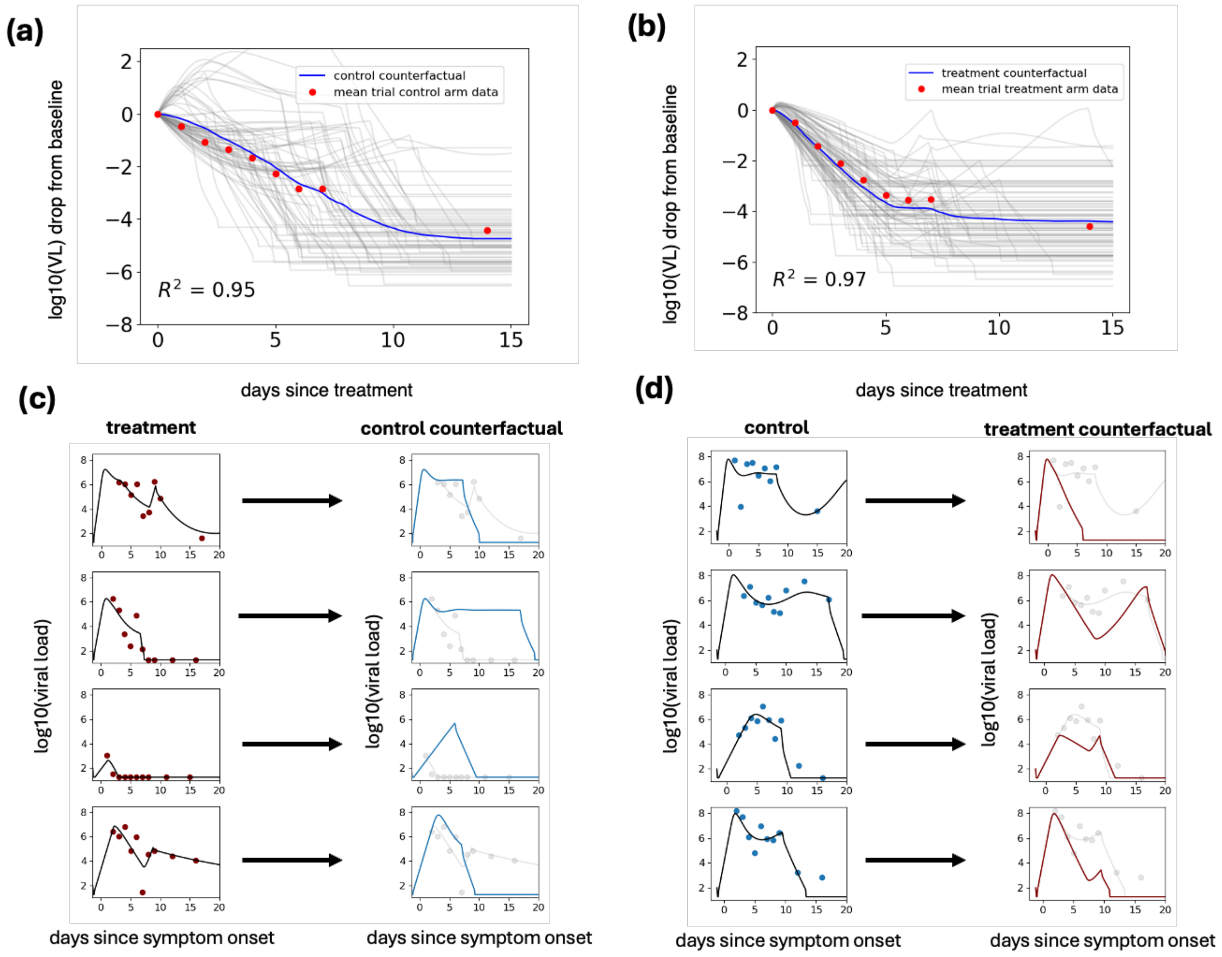

Figure S7. Counterfactual simulations of the control and treatment arms of the PLATCOV trial recapitulate trial outcomes. (a) Simulated control counterfactual of the treatment arm overlayed on control arm trial data. (b) Simulated treatment counterfactual of the control arm overlayed on treatment arm trial data. (a-b) *For each trial,* trial *data are shown in red*, *gray lines are the simulated viral load drop for each individual, and* blue *solid lines are the* simulated *mean viral load drop.* (c) Sample model fits to the treatment arm (model fits in black and treatment arm data in maroon) and its control counterfactual simulation (in blue) (d) Sample model fits to the control arm (model fits in black and treatment arm data in blue) and its treatment counterfactual simulation (in maroon).

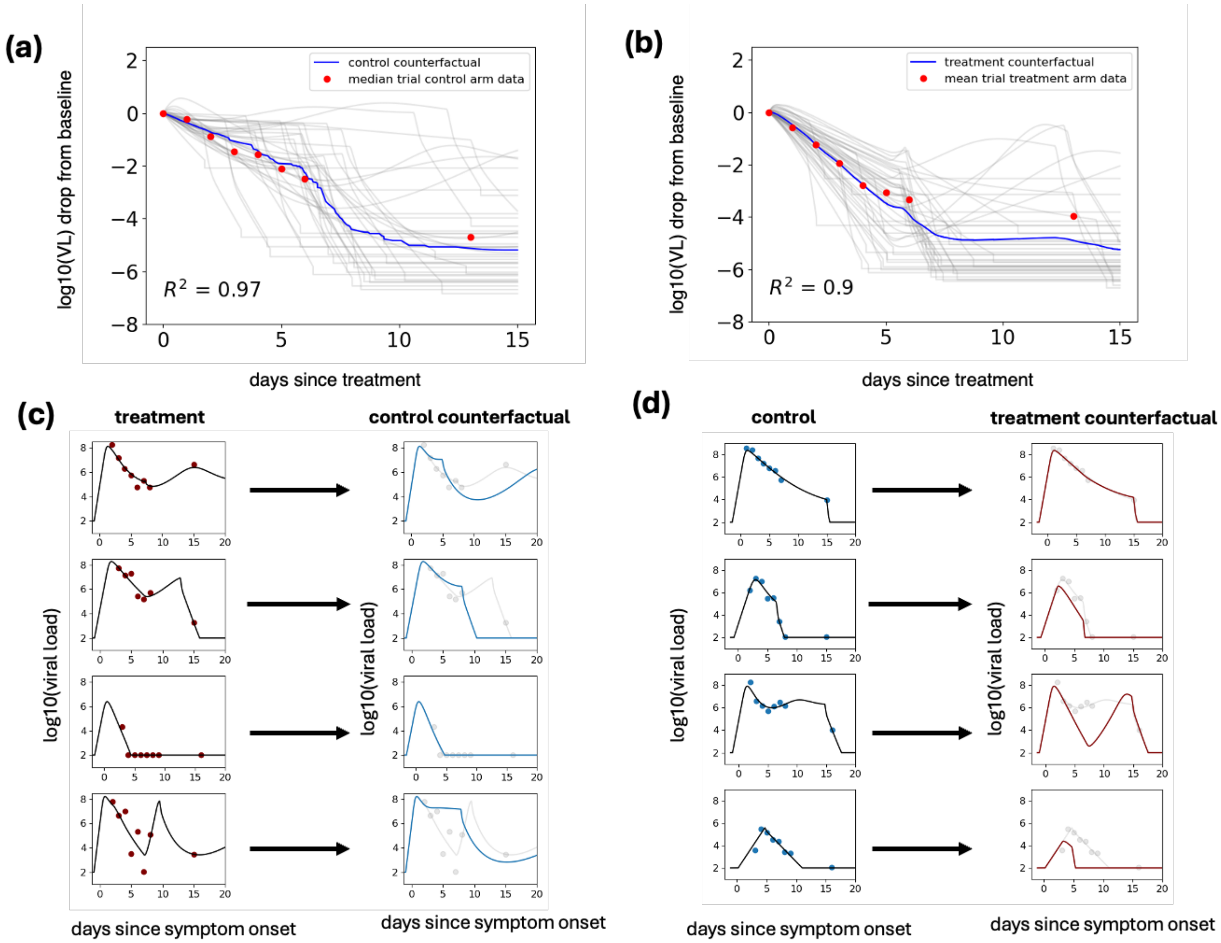

Figure S8. Counterfactual simulations of the control and treatment arms of the PANORAMIC trial recapitulate trial outcomes. (a) Simulated control counterfactual of the treatment arm overlayed on control arm trial data. (b) Simulated treatment counterfactual of the control arm overlayed on treatment arm trial data. (a-b) For each trial, trial data are shown in red, gray lines are the simulated viral load drop for each individual, and blue solid lines are the simulated mean viral load drop. (c) Sample model fits to the treatment arm (model fits in black and treatment arm data in maroon) and its control counterfactual simulation (in blue) (d) Sample model fits to the control arm (model fits in black and treatment arm data in blue) and its treatment counterfactual simulation (in maroon).

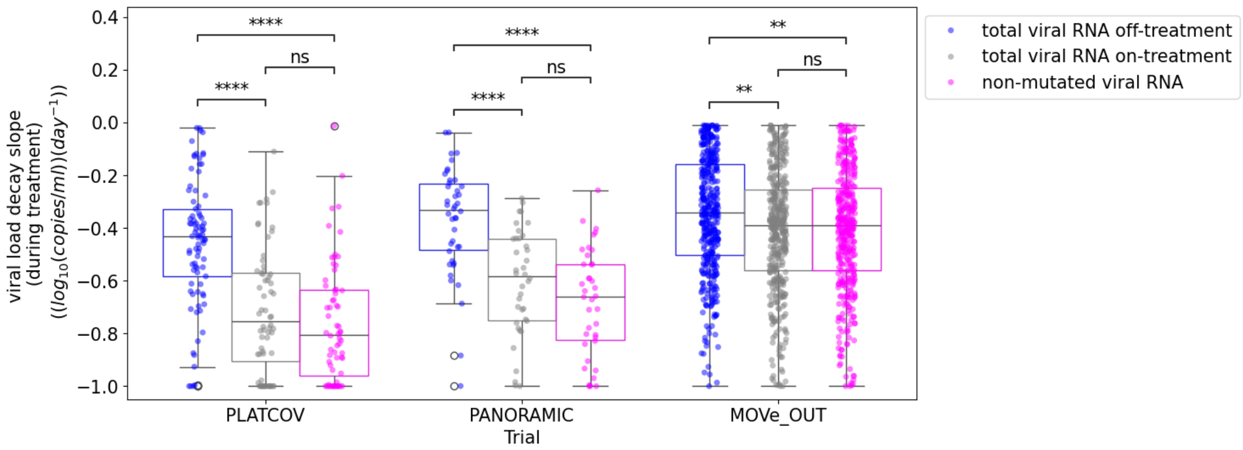

Figure S9. Comparing the viral decay slope of total viral RNA on and off-treatment and non-mutated viral RNA in three trials. There is no significant difference between the rate of decay of total viral RNA on-treatment and non-mutated viral RNA.

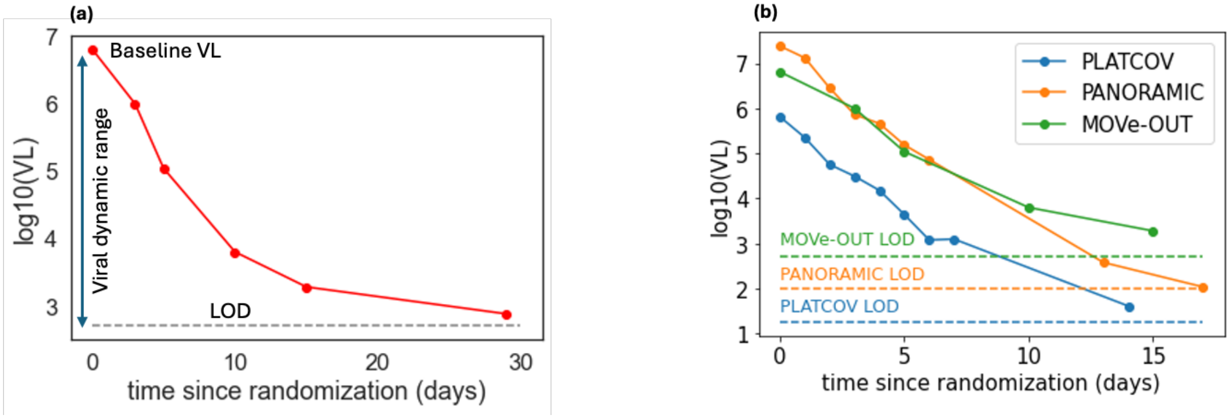

Figure S10. Different viral dynamic ranges in the three trials. (a) Viral dynamic range is defined as the distance from the baseline to the limit of detection (LOD). (b) Comparing the viral dynamic range of the three trials. The dashed lines mark the assay limit of detection (LOD) of each trial.

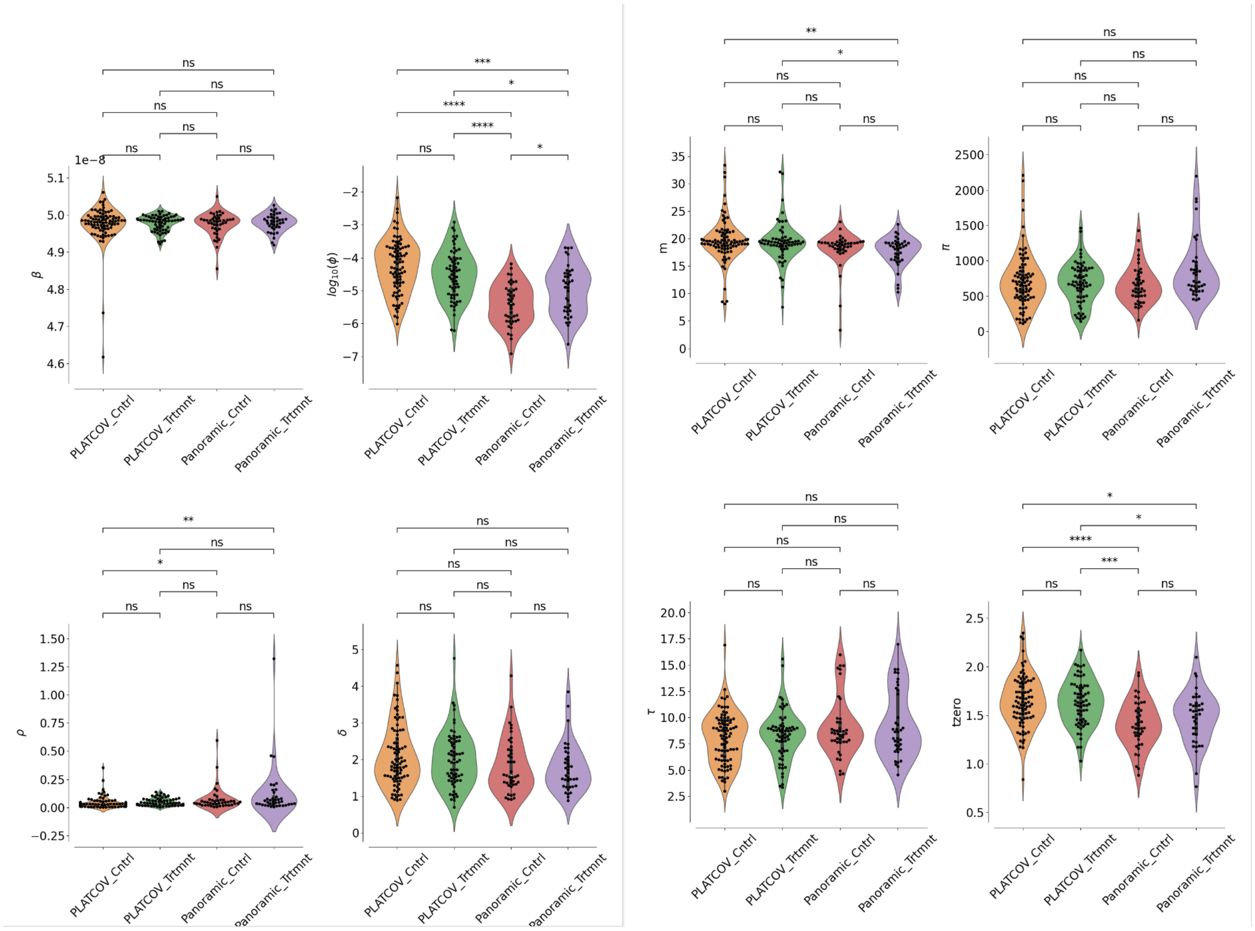

Figure S11. Population parameter distributions two arms of the PLATCOV and PANORAMIC trials. p-values were obtained by performing two-sided Mann-Whitney U-test ($*$: $0.01 < p \leq 0.05$,$**$: $0.001 < p \leq 0.01$,$***$: $0.0001 < p \leq 0.001$,$****$: $0.00001 < p \leq 0.0001$).

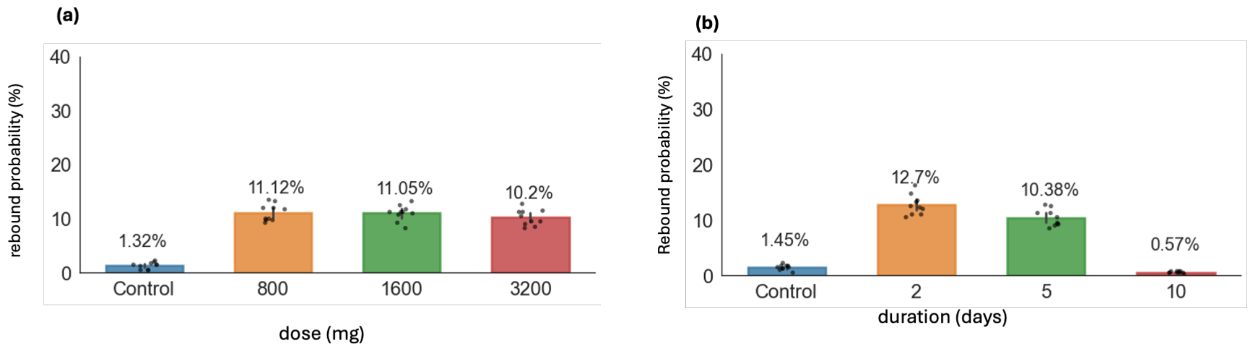

Figure S12. Longer treatment limits the chance of rebound. (a) rebound probability for different doses of molnupiravir administered twice daily for 5 days. (b) rebound probability following 800mg of molnupiravir given twice daily for different durations.

**Dose (mg)**	κ_a*(day⁻¹)*	κ_{LP}*(day⁻¹)*	κ_{CL}*(day⁻¹)*	$\kappa_{PL,1}(mg^{-1})$	**Vol** *(ml)*	α
50	*24.38*	*1.32*	*18.98*	*330.31*	*133637.08*	*-1.01*
100	*23.87*	*1.31*	*19.09*	*325.60*	*134136.89*	*-1.01*
200	*24.41*	*1.3*	*19.18*	*325.70*	*134947.57*	*-1.01*
300	*21.79*	*1.3*	*19.06*	*324.84*	*133957.75*	*-1.01*
400	*22.75*	*1.3*	*19.07*	*326.64*	*134158.16*	*-1.01*
600	*16.71*	*1.31*	*18.91*	*323.64*	*133107.91*	*-1.01*
800	*20.16*	*1.31*	*19.03*	*324.14*	*133791.45*	*-1.01*

Table S1. Estimated PK parameters. Parameters were estimated by fitting PK model to mean plasma concentration. The transition rate between the plasma and peripheral compartment was dose dependent following a powerlaw relationship ($\kappa_{PL} = \kappa_{PL,1} * Dose^{\alpha}$). The parameters were estimated using mixed-effect population approach in monolix.

Parameter (unit)	*Symbol*	*Mean*	*Standard Error*
Maximum efficacy (%)	E_{max}	*100*	*NA*
Drug concentration to provide 50% efficacy ($nMol$ *)*	EC_{50}	*177*	*6.6*
Hill coefficient	n	*2.05*	*0.22*

Table S2. Estimated PD parameters by fitting to in-vitro assay data, using least square method.

Trial	*Clearance half lives in hours: median [IQR]*		
	total viral RNA off-treatment	***Total viral RNA on-treatment***	***Non-mutated viral RNA***
PLATCOV	*16.76, [12.39, 22.07]*	*9.59, [8.01, 12.68]*	*8.97, [7.52, 11.38]*
PANORAMIC	*21.82, [14.94, 31.16]*	*12.38, [9.62, 16.42]*	*10.94, [8.76,13.46]*
MOVe-OUT ***(virtual cohort)***	*21.21, [14.34, 45.52]*	*18.57, [12.90, 28.37]*	*18.5, [12.90, 29.33]*

Table S3. Simulated viral clearance half-life for each trial for total viral RNA on and off treatment and non-mutated viral RNA on treatment. Median clearance half-life ($t_{1/2} = log_{10}(0.5) / clearance\ slope$) for total viral RNA off treatment and on treatment in PLATCOV was 15.5 and 11.6 hours respectively, within the IQR range and within 15 hours of the model estimates for PLATCOV.

Parameter (unit)	Symbol	Population Mean	Standard error	Std dev. of random effects	Distribution	Source
viral infectivity ($\log_{10}$ (RNAcopies/mL)$^{-1}$ day^{-1})	$\log_{10}\beta$	-7.3	2.5e-3	2.5e-2	normal	estimated
viral production rate ($\log_{10}$ day^{-1})	$\log_{10}\pi$	2.77	1.13e-2	0.33	normal	estimated
rate at which refractory cells revert to susceptible state ($\log_{10}$ day^{-1})	$\log_{10}\rho$	-1.47	3.16e-2	0.79	normal	estimated
rate constant for conversion of target cells to a refractory state ($\log_{10}$ cell^{-1}day^{-1})	$\log_{10}\phi$	-4.99	3.86e-2	1.16	normal	estimated
infected cell clearance rate (day^{-1} cells^{-1})	δ	1.64	3.33e-2	0.52	lognormal	estimated
onset of acquired immunity relative to detection (days)	τ	8.34	0.39	0.41	lognormal	estimated
Deviation from τ for unvaccinated/no record	$\beta_{\tau\_>1vax}$	-0.008	3.5e-2	--	--	--
Deviation from τ for NBA Omicron Individuals	$\beta_{\tau_{PLAT,PAN}}$	0.162	3.4e-2	--	--	--
Increase in clearance rate of infected cells due to acquired immunity (day^{-1})	m	19.08	0.76	0.54	lognormal	estimated
time of infection relative to first detection (days)	t_0	1.57	0.09	0.47	logit[0,20]	estimated
Deviation from t_0 for NBA	$\beta_{t_0,PLAT,PAN}$	0.33	6.6e-2	--	--	--
Potency reduction factor for PLATCOV treatment arm	$\log_{10}$paf	-0.79	0.099	0.54	lognormal	estimated
initial viral inoculum (RNAcopies/mL)	V_0	97		--	--	fixed
viral clearance rate (day^{-1})	γ	15		--	--	Goyal et al.
mean eclipse phase duration (days^{-1})	$1/k$	1/4		--	--	Ke et al.
Initial number of susceptible cells	S_0	1×10^7		--	--	Ortiz et al.
Initial number of refractory cells	R_0	0		--	--	--
Initial number of productively infected cells	$I_{P,0}$	0		--	--	--
Initial number of infected cells in eclipse phase	$I_{E,0}$	0		--	--	--

Table S4. Population parameters of combined viral dynamics + PKPD model fit to PANORAMIC+PLATCOV + NBA Omicron data. Using the viral dynamic+PKPD model we estimated model parameters for Omicron infections in the NBA cohort and control and treatment arms of PLATCOV trial. For the mixed-effect model, a constant error model was used, and the magnitude of measurement error was fixed at a = 0.4 log10 copies viral RNA/ml. Vaccine status (0 vs >1 dose) was set as a covariate on τ, and the cohort (NBA vs PLATCOV and PANORAMIC) was set as a covariate for τ and t_0. Also, linear dependencies between (π, ϕ, δ) and (τ, ρ) were set up in Monolix. The population parameters are recorded here and estimated individual parameters are available at https://github.com/sEsmaeili/MolnupiravirModeling.
